# Axial Tubule Junctions Activate Atrial Ca^2+^ Release Across Species

**DOI:** 10.3389/fphys.2018.01227

**Published:** 2018-10-08

**Authors:** Sören Brandenburg, Jan Pawlowitz, Funsho E. Fakuade, Daniel Kownatzki-Danger, Tobias Kohl, Gyuzel Y. Mitronova, Marina Scardigli, Jakob Neef, Constanze Schmidt, Felix Wiedmann, Francesco S. Pavone, Leonardo Sacconi, Ingo Kutschka, Samuel Sossalla, Tobias Moser, Niels Voigt, Stephan E. Lehnart

**Affiliations:** ^1^Heart Research Center Göttingen, Department of Cardiology and Pneumology, University Medical Center Göttingen, Göttingen, Germany; ^2^Heart Research Center Göttingen, Institute of Pharmacology and Toxicology, University Medical Center Göttingen, Göttingen, Germany; ^3^Department of NanoBiophotonics, Max Planck Institute for Biophysical Chemistry, Göttingen, Germany; ^4^European Laboratory for Non-Linear Spectroscopy and National Institute of Optics (INO-CNR), Sesto Fiorentino, Italy; ^5^Institute for Auditory Neuroscience and InnerEarLab, University Medical Center Göttingen, Göttingen, Germany; ^6^Department of Cardiology, University Hospital Heidelberg, Heidelberg, Germany; ^7^DZHK (German Centre for Cardiovascular Research) partner site Heidelberg/Mannheim, University of Heidelberg, Heidelberg, Germany; ^8^Heidelberg Center for Heart Rhythm Disorders, University Hospital Heidelberg, Heidelberg, Germany; ^9^Department of Physics, University of Florence, Florence, Italy; ^10^Department of Cardiothoracic and Vascular Surgery, University Medical Center Göttingen, Göttingen, Germany; ^11^DZHK (German Centre for Cardiovascular Research) partner site Göttingen, Göttingen, Germany; ^12^BioMET, The Center for Biomedical Engineering and Technology, University of Maryland School of Medicine, Baltimore, MD, United States

**Keywords:** atria, atrial myocyte, axial tubule, calcium, heart, ryanodine receptor

## Abstract

**Rationale:** Recently, abundant axial tubule (AT) membrane structures were identified deep inside atrial myocytes (AMs). Upon excitation, ATs rapidly activate intracellular Ca^2+^ release and sarcomeric contraction through extensive AT junctions, a cell-specific atrial mechanism. While AT junctions with the sarcoplasmic reticulum contain unusually large clusters of ryanodine receptor 2 (RyR2) Ca^2+^ release channels in mouse AMs, it remains unclear if similar protein networks and membrane structures exist across species, particularly those relevant for atrial disease modeling.

**Objective:** To examine and quantitatively analyze the architecture of AT membrane structures and associated Ca^2+^ signaling proteins across species from mouse to human.

**Methods and Results:** We developed superresolution microscopy (nanoscopy) strategies for intact live AMs based on a new custom-made photostable cholesterol dye *and* immunofluorescence imaging of membraneous structures and membrane proteins in fixed tissue sections from human, porcine, and rodent atria. Consistently, in mouse, rat, and rabbit AMs, intact cell-wide tubule networks continuous with the surface membrane were observed, mainly composed of ATs. Moreover, co-immunofluorescence nanoscopy showed L-type Ca^2+^ channel clusters adjacent to extensive junctional RyR2 clusters at ATs. However, only junctional RyR2 clusters were highly phosphorylated and may thus prime Ca^2+^ release at ATs, locally for rapid signal amplification. While the density of the integrated L-type Ca^2+^ current was similar in human and mouse AMs, the intracellular Ca^2+^ transient showed quantitative differences. Importantly, local intracellular Ca^2+^ release from AT junctions occurred through instantaneous action potential propagation via transverse tubules (TTs) from the surface membrane. Hence, sparse TTs were sufficient as electrical conduits for rapid activation of Ca^2+^ release through ATs. Nanoscopy of atrial tissue sections confirmed abundant ATs as the major network component of AMs, particularly in human atrial tissue sections.

**Conclusion:** AT junctions represent a conserved, cell-specific membrane structure for rapid excitation-contraction coupling throughout a representative spectrum of species including human. Since ATs provide the major excitable membrane network component in AMs, a new model of atrial “super-hub” Ca^2+^ signaling may apply across biomedically relevant species, opening avenues for future investigations about atrial disease mechanisms and therapeutic targeting.

## Introduction

As one billion individuals 65 years or older are expected by the year 2030, aging populations will be affected by a sharp increase in chronic diseases (Moslehi et al., 2012). Most frequent in elderly people, electrical and contractile dysfunction of the atria contributes to stroke, heart failure, and atrial fibrillation the latter alone predicted to increase threefold in prevalence by 2050 (Yiin et al., 2014). While atrial fibrillation is often considered a proximal cause of thrombembolic stroke, recent clinical studies question a direct relationship (Brambatti et al., 2014; Martin et al., 2015). Therefore, a broader pathophysiological concept of atrial cardiomyopathy was developed, which explicitly addresses atrial myocyte (AM) specific disease mechanisms (Goette et al., 2017). However, given that AM dysfunction represents a central cause of the disease burden, fundamental cellular mechanisms remain unclear (Brandenburg et al., 2016a; Guichard and Nattel, 2017). Recently, an emerging atrial Ca^2+^ nanodomain model, extending significantly beyond the canonical role of transverse tubule (TT) invaginations in ventricular myocytes (VMs), was proposed: super-hub Ca^2+^ signaling based on axial tubule (AT) junctions that rapidly activate Ca^2+^ release *and* atrial contraction through cell-specific molecular nanodomain mechanisms (Brandenburg et al., 2016b).

In ventricular myocytes, TTs occur periodically near sarcomeric Z-lines at a fixed density, which may unify cell-wide Ca^2+^ release (Crossman et al., 2015), and contribute to heterogeneous Ca^2+^ release in heart failure due to TT reorganization (Song et al., 2006; Wagner et al., 2012). In contrast to VMs, AMs are significantly smaller and have significantly different functions [for review (Brandenburg et al., 2016a)], which may explain why few or no cells with TTs were reported in various species including cat (McNutt and Fawcett, 1969; Huser et al., 1996), guinea pig (Forbes and van Neil, 1988), mouse (Forbes et al., 1984), rabbit (Tidball et al., 1991; Maxwell and Blatter, 2017), or rat (Brette et al., 2002; Kirk et al., 2003; Woo et al., 2005; Sheehan et al., 2006; Smyrnias et al., 2010). Notably, denser atrial TT structures were identified in large mammals including sheep (Dibb et al., 2009; Lenaerts et al., 2009), dog (Wakili et al., 2010), pig (Frisk et al., 2014; Gadeberg et al., 2016), cow, horse, and human (Richards et al., 2011). Yet, irrespective of smaller (i.e., rat) or larger (i.e., dog) species, contractile activation occurs generally faster in atrial than ventricular muscle (Lüss et al., 1999), contributing to the atrial ‘kick’ *in vivo*, an essential booster function for ventricular filling and stress adaptation (Brandenburg et al., 2016a).

Since few or no TTs were found in rat AMs, voltage-dependent L-type Ca^2+^ channels (LCC) were thought to function mainly at the surface sarcolemma, where adjacent ryanodine receptor (RyR2) Ca^2+^ release channels were locally activated through peripheral junctions (Thul et al., 2012). Yet in the same rat species, dense or irregular TT morphologies and LCC currents at TT orifices were recently demonstrated (Frisk et al., 2014; Glukhov et al., 2015). In addition to surface-bound locations, we directly counted LCC clusters by superresolution imaging inside mouse AMs, finding a ∼50% higher density in AT than TT components (Brandenburg et al., 2016b). While these observations were only possible through methodological developments that preserve endogenous membrane structures in live AM samples for nanoscopy studies (Wagner et al., 2012, 2014), it is important to note that intact transverse-axial tubule (TAT) networks were confirmed in 100% of isolated AMs (Brandenburg et al., 2016b). Hence, the notion that AMs from small rodent hearts are mostly devoid of excitable TAT structures warrants re-evaluation across species to identify common mechanisms of atrial excitation-contraction coupling. Here, we show in cells and tissues from mouse, rat, rabbit, pig, and human atria that ATs represent the major TAT component and activate Ca^2+^ release instantaneously in AMs. Thus, the specific TAT membrane network architecture of AMs is conserved across commonly investigated species, opening avenues for future interventions in genetically tractable hearts as well as therapeutic studies that elucidate atria-specific disease mechanisms.

## Results

### Mouse, Rat, and Rabbit Atria Contain Cell-Wide TAT Networks

**FIGURE 1:**
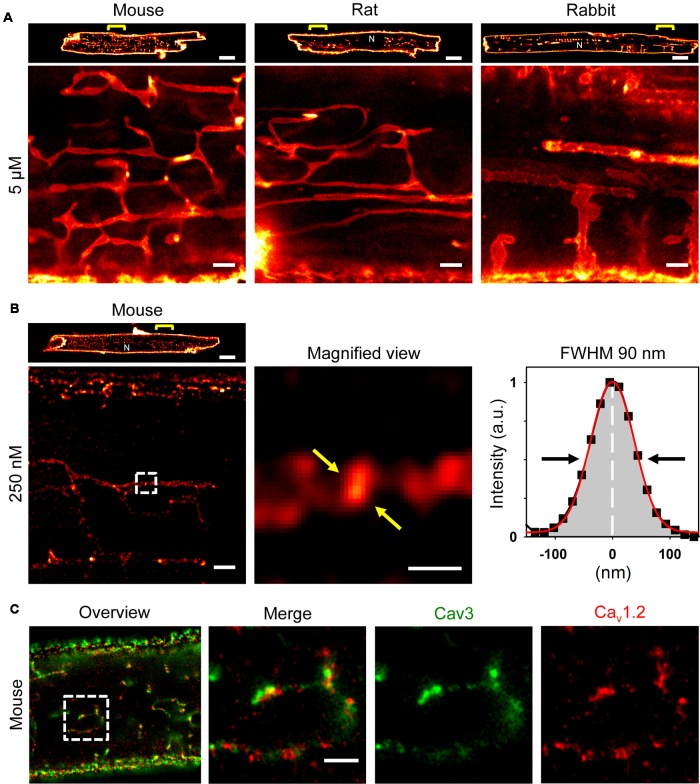
Atrial myocytes from mouse, rat, and rabbit show cell-wide TAT networks composed of abundant axial tubules. **(A)** Live cell imaging of TAT membranes in isolated AMs using Chol-PEG-KK114 as bulk label (5 μM) for STED nanoscopy. Note, abundant axial tubules (ATs) but sparse transverse tubules (TTs) connected with the lateral surface membrane. Examples are representative for three independent AM isolations from three hearts of each species. Scale bars 10 μm (top row) or 1 μm (bottom row). **(B)** Here, the Chol-PEG-KK114 marker concentration was reduced 20-fold (250 nM). Note the clearly delimited cholesterol domains visualized live inside TAT membranes of a mouse AM. The signal intensity distribution of one cholesterol domain (black squares indicate individual data points) was fitted by a Gaussian (red curve) to determine the FWHM ∼90 nm (arrows). Scale bars: top 10 μm; bottom: overview 1 μm; magnified view 200 nm. **(C)** Co-immunofluorescence STED images show Caveolin3 and Ca_v_1.2 clusters in a mouse AM. Scale bar 1 μm. N, nucleus. Yellow brackets and dashed boxes indicate magnified regions.

Recently, we showed that ATs contain a high density of Caveolin3 clusters (Brandenburg et al., 2016b), a bona fide cholesterol binding protein. Hence, we took advantage of a recent strategy to synthesize photostable, far-red emitting fluorescent cholesterol analogs (Chol-PEG-KK114; see methods) for live cell imaging based on STimulated Emission Depletion (STED) nanoscopy (Kolmakov et al., 2010; Sezgin et al., 2016). Bulk labeling with 5 μM Chol-PEG-KK114 readily revealed intact TAT structures in living AMs clearly visible as membrane networks throughout the cytoplasm except the nucleus and continuous with the outer surface sarcolemma in mouse, rat, and rabbit AMs (**Figure 1A**). Notably, while few TTs were visible at the peripheral (cortical) surface membrane, numerous prominent AT components were observed deep inside AMs (**Figure 1A**, magnifications).

In addition to the heart weight (**Supplementary Figure S1A**), the dimensions of AMs vary considerably between mouse, rat, and rabbit (**Supplementary Figures S1B,C**). For example, as compared to mouse AMs, rabbit AMs were significantly longer and wider (**Supplementary Figures S1B,C**). Accordingly, the calculated cell area and heart weight were both correlated in the order mouse < rat < rabbit (**Supplementary Figures S1A,C**). Whereas AMs from the different rodent species differed significantly in their cell dimensions, remarkably, we observed similar cell-wide TAT network morphologies mainly comprised of AT components in isolated AMs.

To explore the vulnerability of TAT membranes to increased cell isolation stress, we performed the same protocol without *in vivo* heparin pre-treatment followed by isolation of rabbit AMs (**Supplementary Figure S2A**). As hypothesized, the TAT network appeared disrupted while residual AT and TT components, visualized by STED nanoscopy, showed locally demarcated membrane fragmentation events, i.e., along AT structures in rabbit AMs (**Supplementary Figure S2B**). In addition, larger aggregates of disrupted membrane components remained stably associated with residual intact membrane structures (**Supplementary Figure S2C**). These results document the vital role of heparin pre-treatment presumably to prevent blood clotting, maintain tissue perfusion, and allow for efficient extracellular matrix digestion via collagenase perfusion. In summary, live cell STED nanoscopy through visualization of individual membrane components can directly discriminate between intact *versus* disrupted individual TAT components in live AMs.

### Cholesterol-Rich Nanodomains Visualized in Intact AT Membranes

While immunofluorescence imaging established that VMs express Caveolin3 throughout TTs, abundant Caveolin3 clusters were identified in AT structures recently (Brandenburg et al., 2016b). Yet we and others have previously shown in VMs that TTs rarely contain caveolae-shaped membrane structures (Wagner et al., 2012; Burton et al., 2017). To identify intact native cholesterol-rich domains in ATs, we adjusted the labeling strategy. Strikingly, a 20-fold lower Chol-PEG-KK114 dye concentration (250 nM) directly resolved individual cholesterol-rich domains deep inside AMs (**Figure 1B**, *left*). While the overall abundance of cholesterol-rich membrane domains was similar on ATs as compared to the caveolae-rich lateral AM surface membrane, STED resolved individual cholesterol domains on ATs (**Figure 1B**, *magnification*). To estimate the size of individual cholesterol domains on a given AT structure, we plotted the intensity profile through a clearly demarcated signal spot and fitted a Gaussian function, resulting in a width (FWHM) of ∼90 nm (**Figure 1B**, *right*). Thus, intact cholesterol-rich domains inside AMs are not only abundant on ATs, but their dimensions are consistent with those of signaling nanodomains.

### ATs Contain Large L-type Ca^2+^ Channel Clusters at Cholesterol-Rich Domains

Depletion of membrane cholesterol was previously shown to decrease the density of caveolae as well as ion conduction through LCCs at the surface of rat AMs (Glukhov et al., 2015). To confirm the expected association between Ca_v_1.2 channels and cholesterol-rich AT nanodomains, we used co-immunofluorescence staining and dual-color STED nanoscopy. Individual Caveolin3 positive signal areas were visualized on AT structures yet located immediately adjacent to Ca_v_1.2 LCC clusters (**Figure 1C**, *left panels*). Since Ca_v_1.2 and Caveolin3 clusters on AT structures were located next to each other, only a minor fraction of the signal overlapped (**Figure 1C**, *merge*). Interestingly, Ca_v_1.2 and Caveolin3 clusters on ATs were relatively large when visualized individually through separate color channels (**Figure 1C**, *right panels*). Taken together, cluster-cluster interactions rather than co-localization of Ca_v_1.2 and Caveolin3 protein complexes on ATs may indicate a key role of cholesterol-rich domains for local LCC regulation of Ca_v_1.2 channel clusters.

**FIGURE 2:**
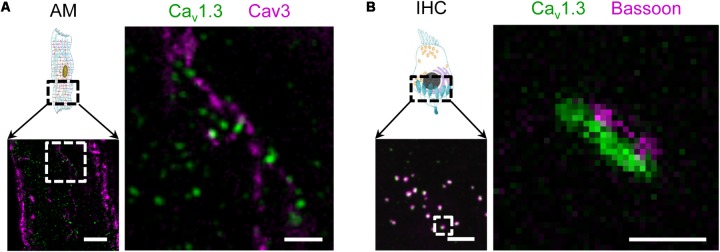
Surface expression of the LCC channel isoform Ca_v_1.3 in AT structures of AMs and at the basal pole of inner hair cells (IHC). **(A)** Co-immunofluorescence STED imaging of Ca_v_1.3 and Caveolin3 (Cav3) in an isolated mouse AM. *Right*: the magnified region (dashed box) shows Ca_v_1.3 next to Cav3 clusters, the latter indicating AT membrane structures. Scale bars: *left* 2 μm; *right* 500 nm (magnification). **(B)** *Left:* Confocal immunohistochemical imaging of Ca_v_1.3 and the presynaptic scaffold protein bassoon showing a representative section of the IHC basal pole. *Right:* A magnified active zone obtained at higher resolution by STED microscopy. In synapses of IHCs, Ca_v_1.3 channels are typically arranged in linear clusters and juxtaposed with bassoon. Scale bars: *left* 2 μm; *right* 200 nm (magnification).

In addition, functional expression of the Ca_v_1.3 LCC isoform has been demonstrated in mouse atria previously (Zhang et al., 2005). As the physiological role of atrial membrane invaginations was unclear at that time, we wondered if the Ca_v_1.3 isoform is also expressed in AT membranes. Indeed, STED co-immunofluorescence labeling of mouse AMs showed that Caveolin3 clusters in AT structures are located close to Ca_v_1.3 clusters (**Figure 2A**). Interestingly, in immunohistological confocal sections of inner hair cells (IHCs) from the inner ear the same antibody identified Ca_v_1.3 clusters associated with the scaffolding protein bassoon (**Figure 2B**, *left*). STED imaging confirmed the close association between presynaptic bassoon and linearly arranged synaptic Ca_v_1.3 channels at the active zone as described previously (**Figure 2B**, *right*) (Frank et al., 2010; Neef et al., 2018). Thus, while both Ca_v_1.2 and Ca_v_1.3 LCCs are expressed in ATs, only the latter isoform is predominantly found in atrial as compared to ventricular myocytes (Zhang et al., 2005). Due to significantly different biophysical properties and more restricted expression, Ca_v_1.3 LCCs may contribute to the unique cell-specific (patho)physiology of AMs and IHCs (see also discussion).

### TAT Network Analysis Reveals Abundant Axial Tubules Across Species

**FIGURE 3:**
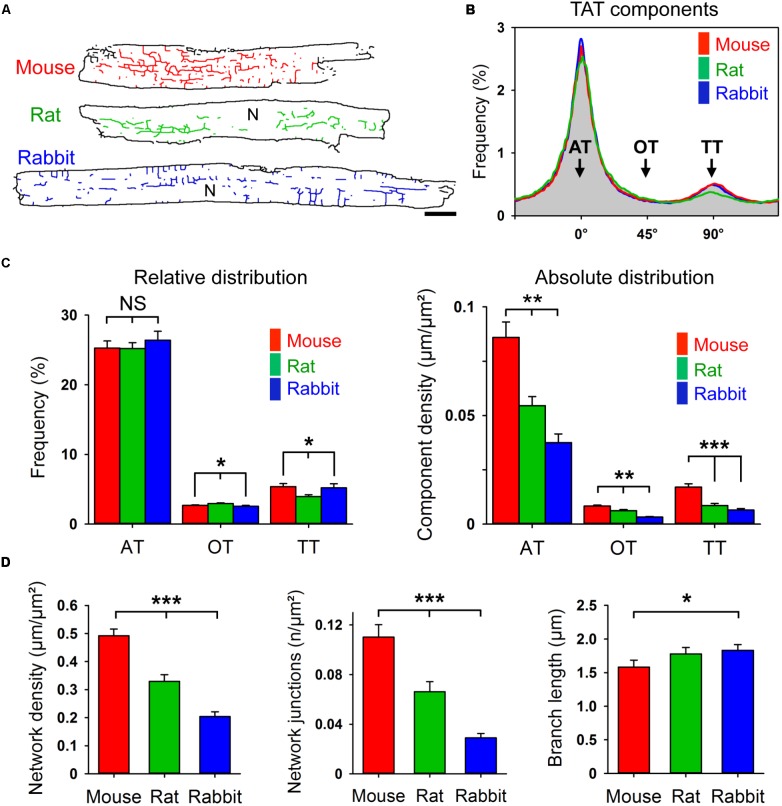
Component-specific TAT analysis in mouse, rat, and rabbit AMs. **(A)** Corresponding TAT live cell images in **Figure 1A** were converted into binarized skeletons for quantitative network analysis. N, nucleus. Scale bar 10 μm. **(B)** The histogram shows the bimodal distribution of specific TAT network components. Major peak: ATs (0°); minor peak: TTs (90°); interim: OTs (oblique tubules, 45°); binning ± 5°. **(C)** Bar graphs comparing the relative and absolute distribution of the three main TAT components. While the relative distribution of TAT components does not differ much between the indicated species, the absolute component density differs significantly between mouse, rat and rabbit AMs. **(D)** The network density, the number of network junctions, and the mean branch length were compared between mouse, rat, and rabbit AM. Cell numbers: 30 mouse, 34 rat, and 23 rabbit AMs, each from 3 individual cell isolations and hearts per species. Student’s *t*-test ^∗^*P* < 0.05, ^∗∗^*P* < 0.01, ^∗∗∗^*P* < 0.001.

Corresponding with **Figure 1A**, live cell images of bulk cholesterol-stained mouse, rat, and rabbit AMs were used for component-specific TAT analysis. For this, the orientation of intact membrane components was analyzed via binarized TAT skeletons oriented according to the major cell axis (**Figure 3A**). Intriguingly, despite the variance in AM and cardiac dimensions between species, orientation-specific TAT network analysis showed similar frequencies of the AT, oblique tubule (OT), and TT components (**Figure 3B**). Importantly, the frequency of the major AT components did not significantly differ between mouse, rat, and rabbit (**Figure 3C**, *left*). For the minor components, only in rat AMs, OTs were more frequent at the cost of TTs (**Figure 3C**, *left*). In contrast, the absolute component densities were significantly different between the species. For example, AT and TT components occurred at a significantly higher density in mouse compared to rat and rabbit AMs (**Figure 3C**, *right*), suggesting an inverse correlation with the calculated cell area (**Supplementary Figure S1C**). While the TAT network density (**Figure 3D**, *left*) and network junctions (**Figure 3D**, *center*) were ranked in the order mouse > rat > rabbit, the mean component branch length was not different between species (**Figure 3D**, *right*). Interestingly, the differences in network density were inversely correlated with atrial cell size (**Supplementary Figure S1C**), and previously correlated with the basal heart rate between different species (Milani-Nejad and Janssen, 2014). Taken together, while mouse, rat, and rabbit AMs showed significant differences in their absolute component and network junction densities, the orientation-specific frequency and absolute density was always dominated by abundant ATs across all rodent species investigated.

### Axial Tubules Have Unique Dimensions

**FIGURE 4:**
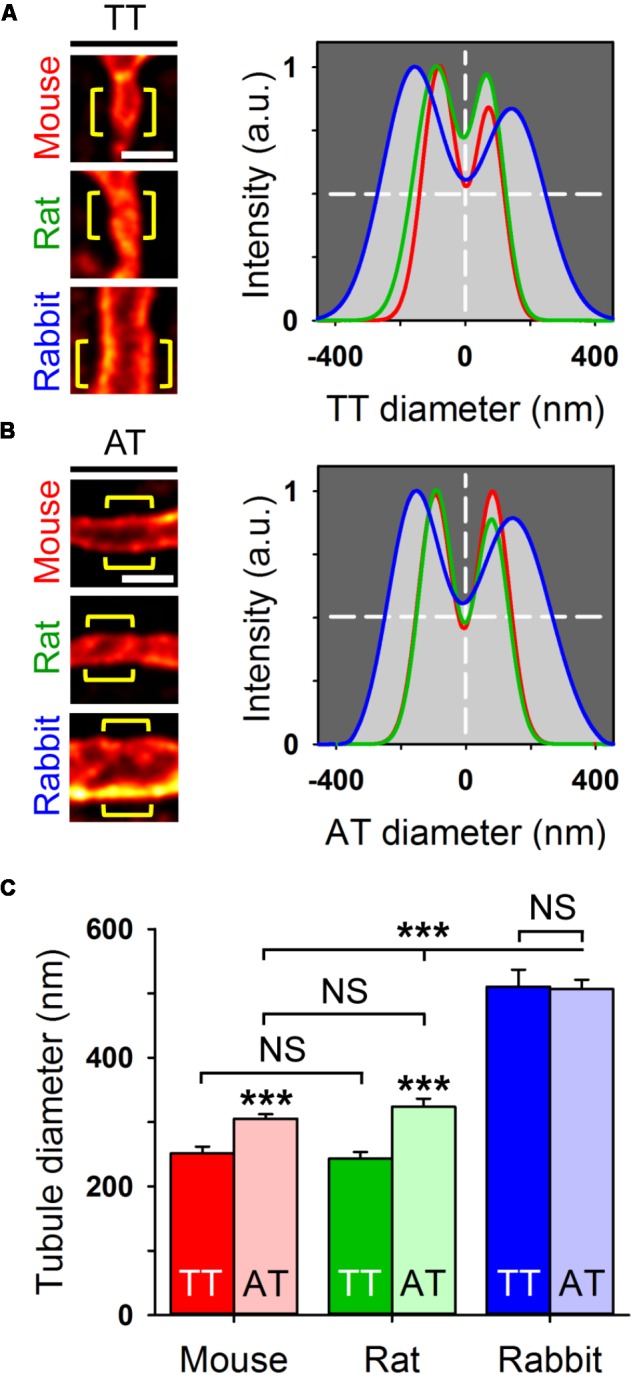
Live cell STED nanoscopy resolves individual tubule dimensions. Representative TT **(A)** and AT **(B)** images from mouse, rat, and rabbit AMs. Bulk staining with Chol-PEG-KK114 (5 μM) and STED nanoscopy resolved the individual tubule membrane structures. Regional intensity profiles from ideally oriented tubule regions (yellow brackets) were used for intensity profiling and Gaussian fitting to estimate tubule width (FWHM) as indicated by horizontal dashed lines. Scale bars in **(A,B)** 500 nm. **(C)** Bar graph comparing TT versus AT diameters (FWHM). In mouse and rat AMs, AT components are significantly larger compared to TT components. In rabbit AMs, AT and TT dimensions far exceed the size of the analogous components in mouse and rat AMs. Component numbers: mouse AM 84 ATs/37 TTs; rat AM 53 ATs/18 TTs; rabbit AM 47 ATs/18 TTs. Data are representative for 3 individual cell isolations and hearts for each species. Student’s *t*-test ^∗∗∗^*P* < 0.001.

In living AMs stained with 5 μM Chol-PEG-KK114, STED nanoscopy resolved the membrane boundaries of individual membrane tubules in the image plane. For analysis, the signal intensity distribution across individual TT components was fitted by a 2-peak Gaussian function, which showed major differences between exemplar mouse, rat, and rabbit tubules (**Figure 4A**). In addition, intensity profiles of AT structures fitted by Gaussian confirmed similar species differences (**Figure 4B**). Importantly, we quantified significantly larger AT than TT widths in mouse and rat AMs (**Figure 4C**), both confirming and extending previous results based on di-8-ANEPPS (Brandenburg et al., 2016b). For example, as compared to TTs, the width of AT components was 18% larger in mouse and 26% larger in rat AMs. In contrast, while the width of AT and TT components was similar in rabbit AMs, it was approximately twice as large compared to mouse and rat AMs (**Figure 4C**). Hence, mouse and rat AMs express significantly larger AT than TT structures.

### Axial Tubule Junctions Contain Extensive, Highly Phosphorylated RyR2 Clusters

**FIGURE 5:**
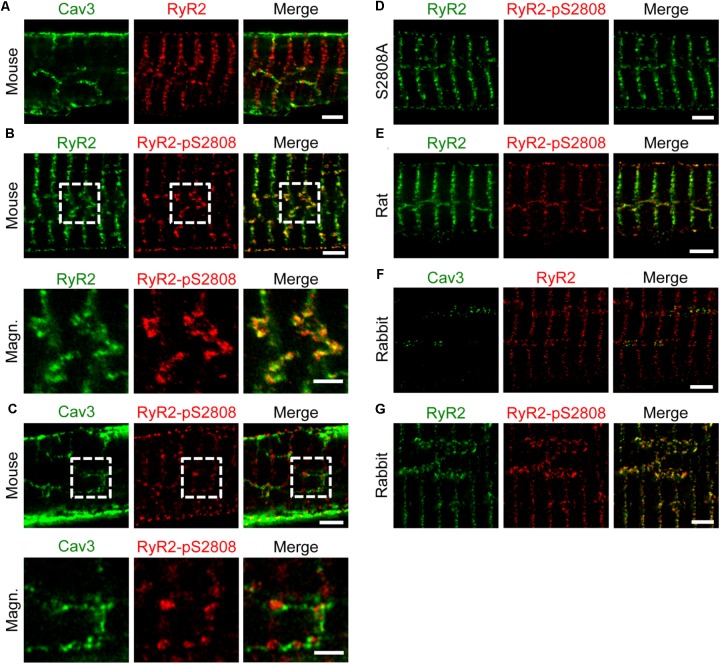
Axial tubules are associated with large, highly phosphorylated RyR2 clusters. STED images show representative AM sections from mouse, rat and rabbit. Co-immunolabeling with primary antibodies against Caveolin3, RyR2 and the phospho-specific epitope RyR2-pS2808 as indicated. **(A)** Caveolin3 labeling is used as a specific marker for the TAT components. Junctional RyR2 clusters are associated with AT structures in contrast to non-junctional RyR2 clusters. **(B)** Through RyR2 and RyR2-pS2808 labeling, large highly phosphorylated RyR2 clusters can be distinguished from low phosphorylated RyR2 clusters. **(C)** The RyR2-pS2808 phospho-epitope specific labeling shows increased PKA phosphorylation of AT-associated junctional RyR2 clusters in contrast to non-junctional RyR2 clusters. **(D)** AM from *Ryr2^S2808A/S2808A^* knockin mice were used as epitope specific negative control. **(E)** Similar axially aligned RyR2-pS2808 clusters were confirmed in rat AM. **(F)** In rabbit AM, larger AT structures align with RyR2 clusters, which **(G)** show increased PKA phosphorylation evidenced by RyR2-pS2808 labeling. Dashed boxes indicate the magnified regions shown below. All scale bars 2 μm, except for magnified regions 1 μm **(B,C)**.

As previously shown by confocal imaging and electron tomography in mouse AMs, ATs form contact junctions of μm extent with the SR, where LCCs can rapidly activate Ca^2+^ induced Ca^2+^ release (CICR) through a less than 15 nm wide subspace (Brandenburg et al., 2016b). Here, immunofluorescence labeling and STED nanoscopy of Caveolin3 and RyR2 in mouse AMs showed RyR2 clusters alternating with Caveolin3 clusters on AT structures (**Figure 5A**). Notably, the majority of RyR2 clusters organized transversally in striations near Z-lines were not associated with any Caveolin3-labeled membrane structures (**Figure 5A**). Additionally, co-immunostaining of RyR2 clusters for RyR2-pS2808 phosphorylation identified highly phosphorylated and axially aligned RyR2 clusters, which intersect the transversally aligned yet minimally Protein Kinase A (PKA) phosphorylated RyR2 clusters (**Figure 5B**, *top*). Indeed, phospho-epitope specific antibody labeling showed that PKA phosphorylated RyR2-pS2808 clusters occurred at AT junctions (**Figure 5C**, *top and bottom*). Confirming earlier confocal studies, the new STED imaging data show specific RyR2 cluster associations and phosphorylation states in AMs, junctional versus non-junctional, where only the former are associated with AT structures.

Importantly, STED nanoscopy further revealed that highly phosphorylated RyR2 clusters appear to be significantly larger compared to less phosphorylated clusters (**Figure 5B**, *bottom*). Moreover, phosphoepitope-specific RyR2-pS2808 labeling was confirmed in PKA-phosphorylation incompetent AMs from *Ryr2*^S2808A/S2808A^ knockin mice through completely ablated RyR2-pS2808 phospho-epitope specific cluster signals (**Figure 5D**). Extending further to rat, again highly phosphorylated RyR2 clusters appear axially aligned, indicating the functional relevance of differential RyR2 cluster phosphorylation in AMs (**Figure 5E**). Finally, rabbit AMs showed axially aligned Caveolin3 and RyR2 clusters (**Figure 5F**), again highly phosphorylated *in situ* at the RyR2-pS2808 residue (**Figure 5G**). In line with the co-immunostaining STED images in mouse, Caveolin3 and RyR2 cluster signals were aligned axially (i.e., with axial tubules), where the clusters alternate spatially but do not co-localize in rabbit AMs. Apparently, and only in rabbit AMs, RyR2 clusters show a much wider lateral separation across AT structures in agreement with our live cell quantification of unusually large AT diameters in rabbit AMs (**Figure 4C**). Hence, across species AMs are characterized by highly phosphorylated RyR2 clusters at AT junctions, in contrast to minimally phosphorylated mostly non-junctional transversal RyR2 clusters near Z-lines.

### A Comparison of Ca^2+^ Currents and Ca^2+^ Transients in Mouse and Human AMs

**FIGURE 6:**
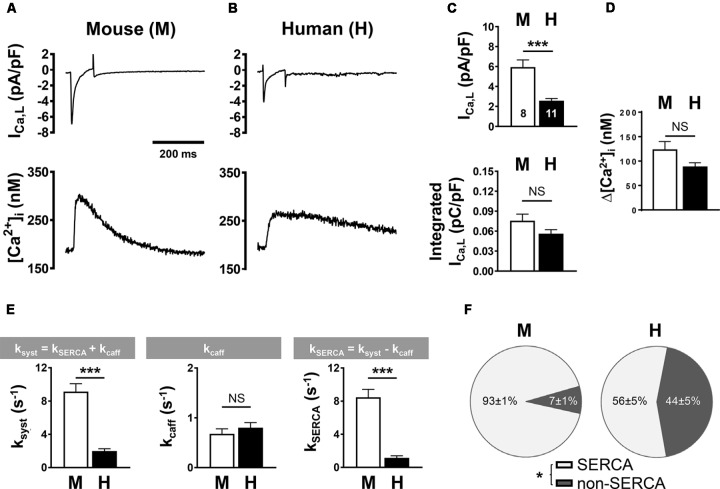
L-type Ca^2+^ current (I_Ca,L_) density and Ca^2+^ transient amplitude in mouse (M) and human (H) atrial myocytes. (**A,B**, *top*) Voltage-clamp was used to depolarize and repolarize the membrane potential at a rate of 0.5 Hz for combined recording of *I*_Ca,L_ and steady-state intracellular Ca^2+^ transients. Representative mouse **(A)** and human **(B)** traces from individual AM recordings are shown. Bar graphs comparing **(C)** the peak (*top*) and the integrated (*lower*) *I*_Ca,L_ density normalized to cell capacitance and **(D)** the systolic Ca^2+^ transient amplitude. **(E)** Bar graphs summarizing the indicated rate constants determined by mono-exponential fitting of systolic Ca^2+^ (k_syst_) and caffeine-induced (k_caff_) Ca^2+^ transients; and the calculated SERCA-dependent rate of decay. **(F)** Pie plots comparing fractional contributions to Ca^2+^ extrusion by SERCA and non-SERCA flux components (NCX, PMCA, and mitochondria) in mouse and human AMs. AMs were isolated from five mouse hearts and six human heart samples. ^∗^*P* < 0.05, ^∗∗∗^*P* < 0.001. Numbers of cells shown in **(C)** apply to all panels. Please refer to **Supplementary Table S1** for clinical information about the human samples.

To compare systolic CICR mechanisms between mouse and human AMs, we simultaneously recorded the L-type Ca^2+^ current (*I*_Ca,L_) and steady-state intracellular Ca^2+^ transients using previously established protocols (Voigt et al., 2013, 2014). Specifically, mouse and human AMs were patch-clamped using the same extracellular Ca^2+^ concentration (1 mM). Despite potential differences in cell size, measurements of the membrane capacitance of isolated mouse and human AMs showed no significant difference (77.07 ± 6.13 pF, *n* = 8; 106.45 ± 15.85 pF, *n* = 11, respectively). *I*_Ca,L_ current traces indicated apparent differences between mouse and human AMs (**Figures 6A** vs. **6B**, *top*). When we calculated the density of *I*_Ca,L_ (**Figure 6C**), the current amplitude normalized to capacitance was significantly smaller in human AMs (*top*) while the integrated *I*_Ca,L_ density showed only a non-significant trend toward lower currents in human AMs (*bottom*). Furthermore, combined Ca^2+^ imaging showed a non-significant decrease of the Ca^2+^ transient amplitude in human compared to mouse AMs (**Figure 6D**).

As membrane capacitance depends both on the outer surface and inner TAT sarcolemma, we explored the state of the TAT membranes in human AMs isolated from relatively small tissue samples of patients (**Supplementary Table S1**). It is important to note that the human AM isolation technique differs significantly from that used for rodent AM isolation in two points: (1) initial mechanical tissue dissection is necessary to provide substrate access for (2) collagenase digestion in sufficiently small tissue pieces in suspension, in contrast to tissue perfusion used in Langendorff hearts (Voigt et al., 2013, 2014). Following isolation via mechanical dissection, the intact state of human AMs stained with 5 μM Chol-PEG-KK114 was documented by bright field and confocal microscopy (**Supplementary Figure S3A**). In addition, STED nanoscopy showed residual AT and TT fragments as well as abnormal membrane aggregates (**Supplementary Figure S3B**). These results confirm that intact human AMs were successfully isolated, while their TAT structures appeared at least partly disrupted. Therefore, we decided to develop an additional tissue-based strategy for structural analysis of the TAT network in human AMs (see further below).

In summary, while these results indicate that membrane capacitance may have been underestimated in human AMs (please refer to the methods section for details) due to partial TAT fragmentation, the integrated *I*_Ca,L_ current and the Ca^2+^ release amplitude in isolated human and mouse AMs were of similar magnitude.

Next, we sought to determine the relative contributions by SERCA and NCX to extrusion of Ca^2+^ from the cytosol during the systolic Ca^2+^ transient. For this, we used experimental protocols that determine the effect of each Ca^2+^ sink individually on the rate of decay (k) of [Ca^2+^]_i_ by fitting systolic and caffeine-evoked Ca^2+^ transients with a single exponential function (O’Neill et al., 1991). **Figure 6E** summarizes the mean data: (*left*) the rate of decay of [Ca^2+^]_i_ during the systolic Ca^2+^ transient was significantly faster in mouse compared to human AMs; (*middle*) while the rate of decay of the caffeine transient was not significantly different (*k*_caff:_ 0.68 ± 0.10 s^−1^ in mouse and 0.80 ± 0.10 s^−^1 in human AMs), the SERCA-dependent rate of cytosolic Ca^2+^ extrusion (k_SERCA_*, right*) was significantly higher in mouse compared to human AMs (*n* = 8 mouse and 11 human cells). Assuming that NCX functions as the predominant Ca^2+^ extrusion pathway, from this analysis the relative contribution to systolic Ca^2+^ extrusion, each by SERCA- and NCX-dependent transport, can be obtained. Whereas the fraction of Ca^2+^ extruded by SERCA was significantly larger in mouse AMs, the fraction extruded by non-SERCA dependent pathways (i.e., NCX) was significantly larger in human AMs (**Figure 6F**).

### Atrial Expression of Ca^2+^ Handling Proteins

**FIGURE 7:**
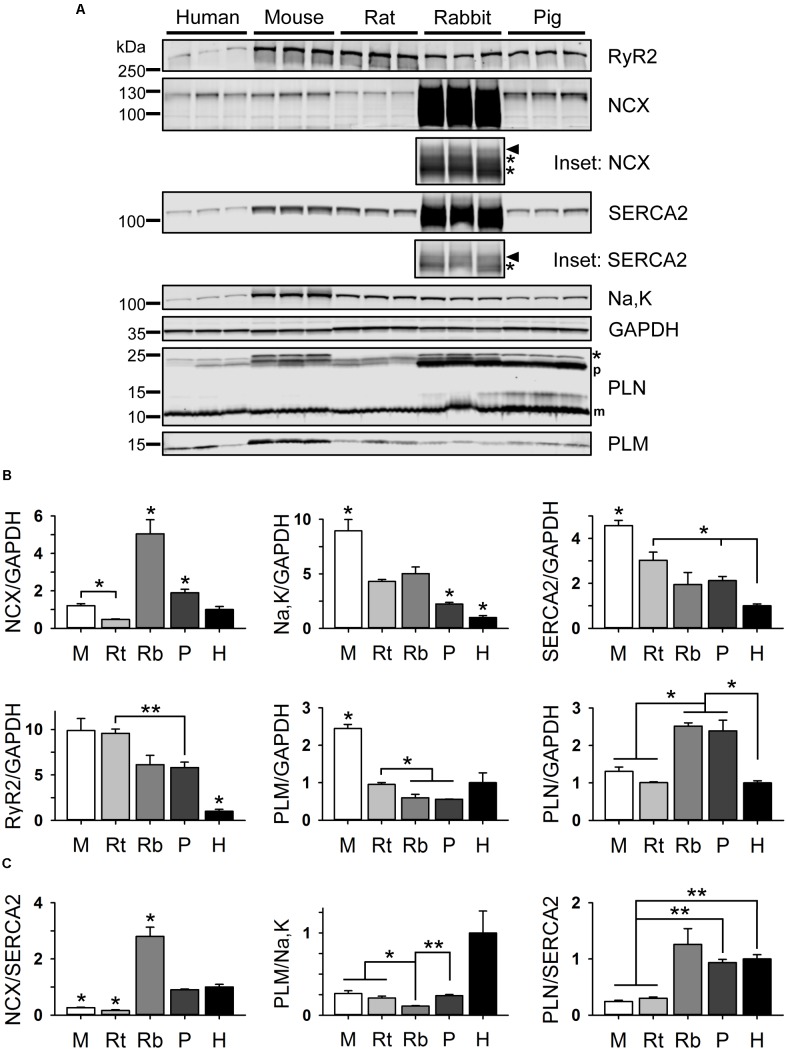
Atrial expression of Ca^2+^ handling proteins in different rodent species, pig, and human. **(A)** Immunoblots comparing the expression of ryanodine receptor 2 (RyR2), sodium-calcium exchanger 1 (NCX), SR Ca^2+^-ATPase 2 (SERCA2), sodium-potassium ATPase α1 subunit (Na,K), glyceraldehyde 3-phosphate dehydrogenase (GAPDH), phospholamban (PLN), and phospholemman (PLM). Insets document the intensity-corrected rabbit NCX and SERCA2 as used for quantification (arrowheads) and additional rabbit-specific antibody cross-reactions (asterisks). Also, the PLN pentameric (p) and monomeric (m) bands are indicated. See **Supplementary Figure S4** for full blots and **Supplementary Table S1** for clinical patient information. Immunoblot/GAPDH signals **(B)** and relative protein ratios **(C)** each normalized to human. M, mouse; Rt, rat; Rb, rabbit; P, pig; H, human. Student’s *t*-test ^∗^*P* < 0.05, ^∗∗^*P* < 0.01.

Previous studies of rat hearts showed that phospholamban (PLN), a physiological inhibitor of the SR Ca^2+^ (SERCA2) pump released by PKA phosphorylation of PLN-S16, is expressed at significantly lower levels in atria compared to ventricles (Walden et al., 2009). In addition, atrial versus ventricular SERCA2 expression was found to be approximately 2-times higher in mouse, rat, and dog (Lüss et al., 1999; Walden et al., 2009; Brandenburg et al., 2016b). Comparing samples from five species (rodents, pig, and human), we analyzed the expression level of the Na^+^/Ca^2+^-exchanger (NCX), the Na^+^/K^+^-ATPase α-subunit (Na,K-ATPase), and SERCA2 by immunoblotting (**Figure 7A**; the full blots are shown in **Supplementary Figure S4**). While human atrial NCX protein levels were not significantly different from mouse and rat, we found significantly higher Na,K-ATPase and SERCA2 levels in mouse atria, gradually decreasing in line with different species-dimensions with the lowest significant levels found in human atria (**Figure 7B**). In addition, expression of the cardiac ryanodine receptor (RyR2) and phospholemman (PLM) was significantly higher in mouse compared to human atria (**Figure 7B**). Importantly, while PLN levels varied across species, in mouse and human atria PLN expression was not significantly different (**Figure 7B**).

As expected for human compared to mouse atria (Lüss et al., 1999; Walden et al., 2009; Brandenburg et al., 2016b), the capacity to extrude Ca^2+^ to the extracellular space versus Ca^2+^ uptake by the SR was significantly higher as indicated by the NCX/SERCA2 ratio (**Figure 7C**). Furthermore, the PLM/Na,K-ATPase ratio tended to be higher in human atria relative to any other species (**Figure 7C**). Finally, the PLN/SERCA2 ratio was significantly smaller in mouse and rat compared to larger species (**Figure 7C**). These differences in protein expression support a higher versus lower dependence on SERCA2 in mouse as compared to human AMs, respectively. In agreement with the protein expression data, in human AMs we found a significantly lower SERCA-dependent rate of Ca^2+^ removal and a greater fraction of Ca^2+^ extrusion by NCX (**Figures 6E,F**).

### Excitation and Ca^2+^ Release at Axial Junctions Occur Instantaneously

**FIGURE 8:**
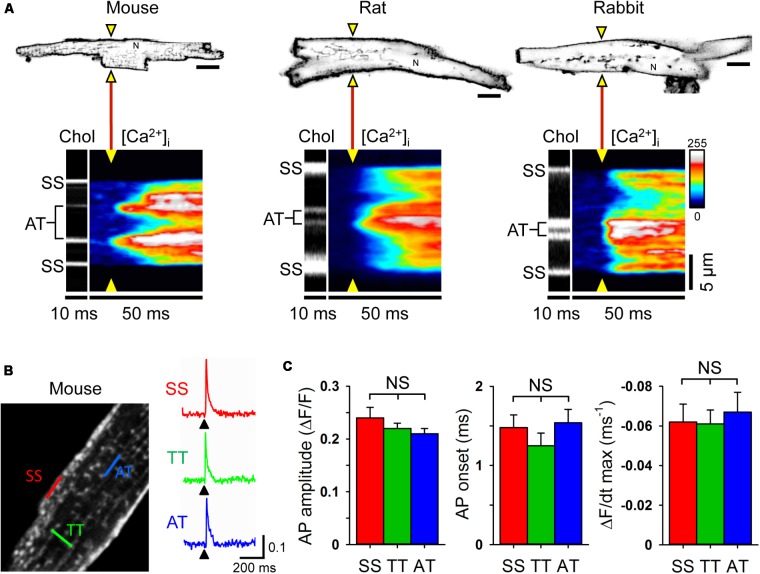
Rapid intracellular Ca^2+^ release at ATs triggered by instantaneous action potential propagation. **(A)** Combined confocal imaging of AT membranes via Chol-PEG-KK114 and [Ca^2+^]_i_ via Fluo-4 in mouse, rat and rabbit AM during 0.5 Hz electrical field stimulation. *Top row:* AM imaging plane and position of the laser line (yellow triangles) used to detect intracellular Ca^2+^ release at AT structures (see also section “Materials and Methods”). *Bottom row:* Transversal line scans showing two ATs localized by Chol-PEG-KK114 (Chol) between the surface sarcolemma (SS) boundaries in representative AMs from each species. Note the earliest local Ca^2+^ transient onset and amplitude dynamics upon electrical field excitation (yellow triangles). N, nucleus. Scale bars top row 10 μm. **(B)** *Left:* Exemplar 2-photon recording from a mouse AM. TAT components were labeled with the voltage-sensitive dye di-4-ANE(F)PTEA (2 μM). *Right:* Normalized fluorescence traces (Δ*F*/*F*_0_) recorded from representative scan regions indicated in color. Simultaneous action potential activation upon stimulation (black arrowheads) is clearly visible in TT and AT structures. **(C)** Grouped bar graphs show no significant differences between AT and other membrane locations for AP amplitude (Δ*F*/*F*), AP onset (the time interval between the end of the stimulus and the rise of the fluorescence signal above a threshold of 4% Δ*F*/*F*), and maximum slope (Δ*F*/dt). Sample numbers: *n* = 13 SS, 44 TT, and 8 AT from 13 AMs and 4 mouse hearts.

Since relatively large LCC clusters are expressed in AT membranes (**Figure 1C**) and AT-localized clusters occur at a high local density (Brandenburg et al., 2016b), upon electrical excitation CICR may rapidly activate directly adjacent, highly phosphorylated RyR2 clusters through AT junctions (**Figures 5B,C**). To visualize CICR locally at AT junctions, we applied transverse line scanning for combined AT membrane localization and high-resolution intracellular Ca^2+^ imaging in mouse, rat, and rabbit AMs using fluo-4 AM and Chol-PEG-KK114 (1 μM) as membrane stain (**Figure 8A**). Visualization of AT structures during electrical excitation showed rapid yet highly heterogeneous Ca^2+^ release, apparently earlier at AT membranes relative to membrane-free non-junctional sites. In addition, while Ca^2+^ release at the surface sarcolemma appeared to be fast, a delay relative to the more rapid signal activation via CICR at AT sites was apparent in mouse, rat, and rabbit AMs (**Figure 8A**, [Ca^2+^]_i_ *transients*). Taken together, excitation rapidly triggers Ca^2+^ release at AT junctions likely through extensive junctional RyR2 clusters that are highly phosphorylated and, thus, more sensitive to activation through CICR.

While ATs represent the most distant network components relative to the outer surface sarcolemma, action potentials are thought to be conducted initially through TTs to downstream membrane network structures. Thus, we wondered if and how action potentials propagate to AT components. To directly visualize the membrane depolarization of AT components, we recorded the local fluorescence signal of the voltage-sensitive dye di-4-ANE(F)PTEA by random access multi-photon microscopy (RAMP). RAMP measurements allowed to record robust fluorescence signals during steady-state electrical field stimulation at 0.34 Hz throughout the surface sarcolemma at distinct TAT structures in mouse AMs (**Figure 8B**). Apparently, upon field stimulation, action potential depolarization occurred simultaneously at ATs relative to other membrane sites (**Figure 8B**). Analysis of the action potential amplitude, AP onset (the time interval between the end of the stimulus and the rise of the fluorescence signal above a threshold of 4% Δ*F*/*F*), and maximal slope did not reveal any significant differences between AT, TT or surface sarcolemmal locations consistent with instantaneous electrical propagation and voltage-dependent activation of CICR throughout AT components (**Figure 8C**). These results underline the tight electrical coupling between the AM surface and its membrane invaginations through a continuous, intact sarcolemmal system and, furthermore, suggest a space constant of the TAT membrane network larger than the cellular dimensions in line with previous findings in ventricular cardiomyocytes (Kong et al., 2017; Scardigli et al., 2017).

### Abundant AT Structures in Human, Porcine and Rodent Atrial Tissues

**FIGURE 9:**
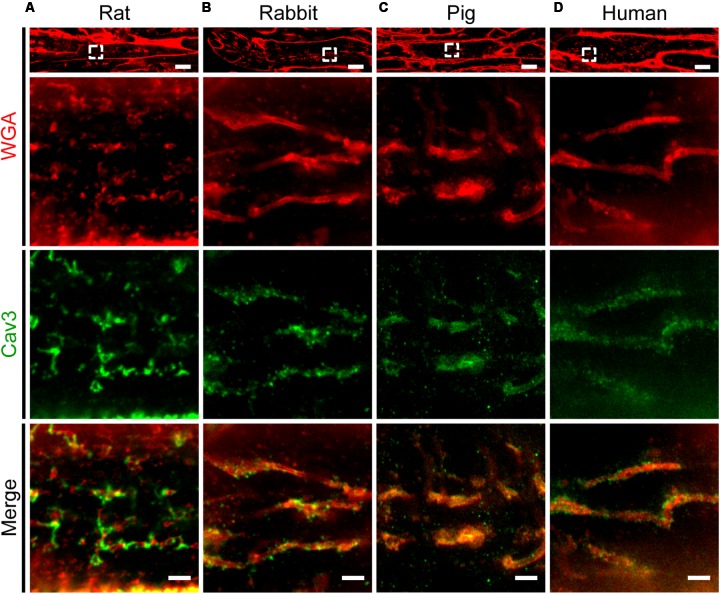
Comparison of TAT structures in rat, rabbit, porcine, and human atrial tissue sections. STED imaging was used to identify individual AMs in **(A)** rat, **(B)** rabbit, **(C)** pig, and **(D)** human atrial tissue co-stained by WGA and Caveolin3 antibody (see methods for details). The magnified regions (dashed boxes) highlight local subcellular structures: WGA signals staining the intraluminal surface of ATs (red), in contrast with punctate Caveolin3 patterns at the abluminal cytosolic AT surface (green). Images are representative of immuno-histological stainings from three animals or patients each. Please see **Supplementary Table S1** for clinical patient information. Scale bars: top 10 μm; magnifications, 1 μm.

Previous studies established protocols to stain TAT components by wheat germ agglutinin (WGA) in histological sections of larger species including human (Richards et al., 2011). Specifically, WGA labels *N*-acetylglucosamine, neuraminic, and sialic acid side chains on the extracellular membrane surface of cardiomyocytes (Arakel et al., 2014). Here, we adapted the WGA staining for paraffin-embedded atrial tissue sections from rat, rabbit, pig, and human atrial samples for STED imaging. In rat atrial tissue sections we observed discrete intracellular WGA signal spots, alternating with Caveolin3 positive domains on TAT structures (**Figure 9A**). In contrast to rat, atrial tissue sections from rabbit and pig showed a more extensive pattern of WGA labeling inside the lumen of AT structures, tightly surrounded by punctate Caveolin3 signals on the abluminal cytosolic surface (**Figures 9B,C**). Importantly, similar to the pattern in rabbit and pig, we identified extensively WGA-labeled AT components in human right atrial tissue sections (**Figure 9D**, *WGA*). Similar to rabbit and pig, discrete abluminal Caveolin3 signals were tightly associated with the AT structures in human atrial sections (**Figures 9B–D**, *merge*).

**FIGURE 10:**
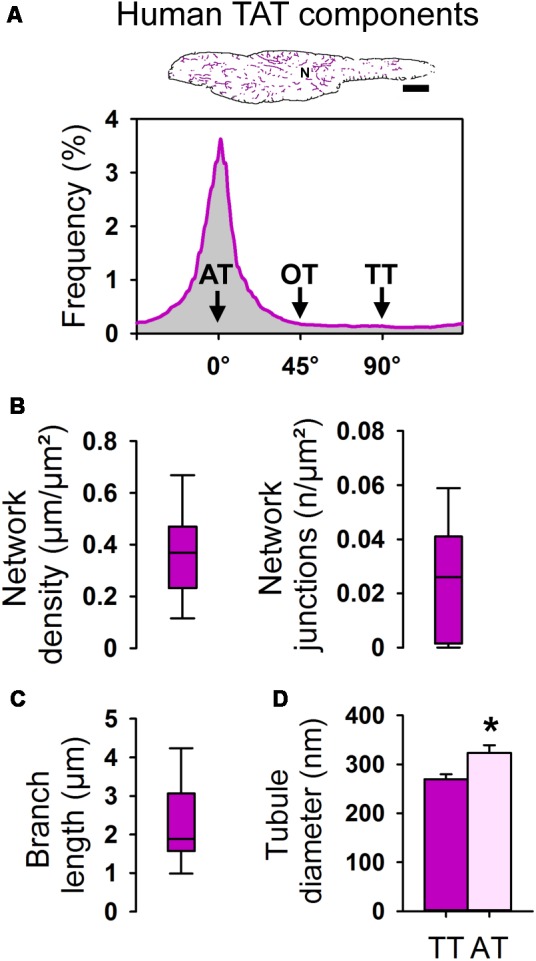
Component-specific TAT analysis of atrial myocytes in human tissue samples. **(A)** Skeleton representation (pink) of the WGA stained human AM from **Figure 9D**. Histogram analysis showing the frequency distribution of individual TAT components. The major peak represents abundant ATs (0°). N, nucleus. Scale bar 10 μm. **(B)** Box plots summarizing the TAT network density and the number of network junctions. **(C)** Box plot showing the branch length in human AM. Boxes represent the median, lower and upper quartiles, and whiskers the 5th and the 95th percentiles. *n* = 21 AMs in histological tissue sections from three patients. **(D)** Bar graph summarizing the AT and TT diameter (FWHM) as determined in WGA stained AMs in human tissue sections. *n* = 35 ATs and 17 TTs from 22 human AMs in tissue sections. Please see **Supplementary Table S1** for clinical patient information. Student’s *t*-test ^∗^*P* < 0.05.

Next, the WGA-labeled TAT network signals, visualized by STED in individual human AMs in tissue sections, were extracted as skeleton information and analyzed as follows. First, screening of human atrial tissue samples for individual AMs with the major longitudinal cell axis in the imaging plane through the central nucleus was used to visualize TAT skeletons (**Figure 10A**, *top*). Second, the skeletonized image information was analyzed to determine the orientation and frequency of individual TAT network components (**Figure 10A**, *bottom*). The frequency histogram clearly shows a major peak confirming abundant AT structures in human AMs of atrial tissue sections. We note that the expected minor peak at 90° due to TT components, as shown in isolated live rodent AMs (**Figure 3B**), was lost, which can be attributed to an increased 3D variability of AM orientations in atrial tissue. Consequently, we studied the nature of individual TT components in transversally cut AMs in atrial tissue sections. Interestingly, while occasional TT invaginations were clearly identified in AM cross-sections in atrial tissue, the path of TTs from the lateral surface membrane was often oblique, indicating additional limitations for the TT specific component analysis in atrial tissue sections (**Supplementary Figure S5**).

In human atrial tissue sections we determined an average TAT network density of 0.368 μm/μm^2^ (median 0.369 μm/μm^2^) and 0.023/μm^2^ (median 0.026 n/μm^2^) for the number of network junctions (**Figure 10B**). The average branch length amounted to 2.29 μm (median 1.88 μm) in human AMs in tissue sections (**Figure 10C**). Finally, *in situ* STED nanoscopy was used to measure the cross-sectional diameters of individual AT and TT components. Strikingly, the width (FWHM) of ATs was significantly larger compared to TTs in human AMs in WGA-stained tissue sections (**Figure 10D**). In summary, human AMs contain TAT networks that consist mainly of voluminous AT structures consistent in width with intact AT structures observed in isolated mouse and rat AMs.

## Discussion

Across five species, from mouse to human, we identify continuous intracellular tubule networks in AMs characterized by: (1) abundant AT structures oriented along the main cell axis and (2) connected to the AM surface through sparse TT components. During electrical stimulation, (3) AP depolarization at ATs was instantaneous relative to the surface sarcolemma and contributed to (4) rapid CICR activation at ATs. In line with a previously proposed model of atrial super-hub Ca^2+^ signaling, we confirmed that (5) AT structures exhibit significantly larger diameters compared with TTs in most species except rabbit; (6) ATs are spatially associated with unusually large RyR2 clusters, and (7) junctional RyR2 clusters are constitutively highly phosphorylated. While these observations present a robust basis to explain AT-dependent, rapid intracellular Ca^2+^ signaling in AMs, they were only possible through membrane-preserving isolation and staining protocols, here visualized through a photostable fluorescent cholesterol dye and live cell STED nanoscopy. Importantly, the concept of AT-dominated TAT networks as atria-specific mechanism of excitation-contraction coupling appears to be conserved throughout the species investigated. In line with the recently discovered physiological significance of ATs for graded intracellular CICR regulation (Brandenburg et al., 2016b), we propose that super-hub Ca^2+^ signaling represents a structurally conserved atrial mechanism that may fundamentally change our perspectives about atrial function through specific subcellular contractile activation mechanisms in health and disease.

In contrast, earlier *and* recent studies could not identify any TAT components in isolated rat, rabbit, or cat AMs (Huser et al., 1996; Brette et al., 2002; Thul et al., 2012; Greiser et al., 2014; Maxwell and Blatter, 2017). Accordingly, the TAT volume ratio quantified in rabbit atrial as compared to ventricular thin sections by electron microscopy was over ∼10-fold lower (Tidball et al., 1991). Together, such imaging data led to influential theories about physiological and disease mechanisms, for example how intracellular Ca^2+^ signaling is altered due to atrial fibrillation or rapid pacing, usually based on models devoid of TTs with slow inward propagated Ca^2+^ transients as reviewed in detail (Bootman et al., 2011; Greiser et al., 2011; Schotten et al., 2011; Heijman et al., 2016; Goette et al., 2017; Greiser, 2017). However, confocal imaging studies also showed clear evidence of TAT networks yet only in fractions of isolated rat AMs, generally attributed to a larger cell width (Kirk et al., 2003; Smyrnias et al., 2010; Johnsen et al., 2013; Glukhov et al., 2015). Furthermore, both AT and TT components were described in atrial EM sections of the mouse and rat (Forssmann and Girardier, 1970; Forbes et al., 1984). Accordingly, dense TAT structures were shown in isolated mouse AMs (Greiser et al., 2014). Finally, atrial TT structures were documented in larger mammalian species including dog (Wakili et al., 2010), sheep (Dibb et al., 2009; Lenaerts et al., 2009), pig (Frisk et al., 2014; Gadeberg et al., 2016), cow, and horse (Richards et al., 2011). Of note, TTs were either absent in isolated human AMs (Greiser et al., 2014) or present in human atrial tissue sections (Richards et al., 2011). While Richards et al. developed a WGA-based protocol for human AMs to assess the TT density, they did not analyze the different tubule components, particularly not ATs in histological tissue sections with longitudinally aligned AMs. Taken together, the nature of the atrial sample (i.e., isolated cell or tissue section), cell isolation or tissue dissection protocols, imaging techniques to identify TAT structures in isolated AMs or atrial tissue, and other factors can contribute significantly to the outcome of quantitative TAT imaging studies.

Here, we developed new strategies for high-resolution imaging of TAT networks in isolated AMs and tissue sections. A bright and photostable fluorophore (i.e., KK114) was linked by a large extracellular PEG domain to cholesterol to prevent dye internalization and stably label the extracellular membrane leaflet. While bulk membrane labeling at higher concentrations (5 μM) showed continuous TAT network structures throughout different species except human, live cell STED nanoscopy using significantly lower concentrations (250 nM) directly resolved cholesterol nanodomains in AT structures (**Figure 1**). Importantly, while continuous tubule signals are generally interpreted as healthy intact membrane structures in confocal images, STED nanoscopy clearly showed local membrane fragmentation events, directly documenting membrane disruptions due to on-purpose suboptimal rabbit AM isolation (**Supplementary Figure S2**). Here, global and local AT fragmentation and membrane aggregation were evidenced in rabbit AMs due to absent heparin pre-treatment. Furthermore, human AMs isolated by mechanical tissue dissection showed only residual AT and TT fragments (**Supplementary Figure S3**), whereas continuous cell-wide TAT components were identified in AMs visualized individually in human tissue sections (**Figure 10A**). Notably, a confocal live imaging study found no TAT structures in isolated human AMs previously (Greiser et al., 2014). Thus, for detailed subcellular TAT studies in AMs, labeling of intact TAT membranes combined with live cell nanoscopy provides a powerful strategy to directly identify intact tubule structures.

Cholesterol accounts for 25–50% of the total lipid content in eukaryotic membranes (Bloch, 1989). Although cholesterol is an abundant membrane component, it is concentrated in cholesterol-rich domains, also known as membrane rafts (Simons and Ikonen, 1997). In VMs cholesterol-rich domains were established in caveolae, where Caveolin3 binds cholesterol with high affinity (Parton and Simons, 2007). Importantly, in AMs we observed AT structures densely populated by cholesterol-rich nanodomains (**Figure 1B**) and frequent Caveolin3 clusters (**Figure 1C**). However, in the TTs of VMs few caveolae-like structures exist (Wagner et al., 2012; Burton et al., 2017) and AT membranes in AMs are also devoid of classic caveolae (Brandenburg et al., 2016b). Moreover, cholesterol-rich domains interact functionally with Ca_v_1.2 LCCs in VMs (Balijepalli et al., 2006) and AMs (Glukhov et al., 2015). Chemical depletion of cholesterol in AMs decreased LCC function in caveolae but not at TT orifices (Glukhov et al., 2015) and altered TTs structurally (Carozzi et al., 2000). Interestingly, while Ca_v_1.2 and Caveolin3 clusters were closely associated, STED nanoscopy showed no major co-localization (**Figure 1C**), which may indicate a modular organization of two directly adjacent nanodomains. Interestingly, in skeletal muscle cells, the highest density of LCCs was determined in TAT membranes (Almers et al., 1981; Jorgensen et al., 1989). Future studies may elucidate the impact of cholesterol-rich nanodomains in AT membranes on local nanodomain functions.

High-resolution scanning of LCC ion conductance (SICM) at the lateral membrane of AMs has shown two major, equally distributed functional surface locations: the membrane crest and TT orifices (Glukhov et al., 2015). While LCCs that contain the pore-forming Ca_v_1.2(α1C) subunit are commonly expressed in cardiomyocytes, expression of the Ca_v_1.3(α1D) isoform is highly selective, for example restricted to AMs and the sinoatrial node (Zhang et al., 2005). In this context, a unique electrophysiological and molecular identity of AMs was uncovered through knockout of the pore-forming Ca_v_1.3(α1D) subunit, which revealed a lower activation threshold compared to Ca_v_1.2(α1C) (Zhang et al., 2005). Interestingly, the SANDD syndrome, diagnosed in patients with sinoatrial node dysfunction and deafness, is caused by genetic defects of the Ca_v_1.3(α1D) isoform (Baig et al., 2011). Previously we showed in mouse AMs that ATs contain a significantly higher number and density of Ca_v_1.2 clusters compared to TTs, suggesting important structural and functional roles of AT junctions during CICR (Brandenburg et al., 2016b). Here, we identified both large Ca_v_1.2 and Ca_v_1.3 LCC clusters on AT structures (**Figures 1C**, **2A**) and adjacent highly phosphorylated RyR2 clusters using STED nanoscopy (**Figure 5**). Finally, the same antibody showed presynaptic Ca_v_1.3 clusters in IHCs isolated from the inner ear of mice (**Figure 2B**). Given that Ca_v_1.3(α1D) knockout in mice is viable (Platzer et al., 2000; Zhang et al., 2005) and reproduces key aspects of the SANDD syndrome, future studies will need to characterize the yet unknown role of Ca_v_1.3 LCCs in ATs and how AM function might be affected by knockout or SANDD mutations.

In addition to voltage-dependent LCC activation, ATs are functionally coupled to unusually large RyR2 clusters in axial couplons, extensive junctional nanodomains that allow for rapid CICR onset (Brandenburg et al., 2016b) and characteristically shape atrial Ca^2+^ transients (**Figure 8A**). STED nanoscopy confirmed extensive RyR2 clusters at AT membranes that were highly phosphorylated by PKA *in situ*, and hence functionally different from directly neighboring minimally phosphorylated non-junctional RyR2 clusters (**Figure 5**). The AM-specific PKA phosphorylation pattern correlated not only with substantially earlier Ca^2+^ release from AT-associated RyR2 clusters (**Figure 8A**), but also with faster central sarcomere shortening as shown previously (Brandenburg et al., 2016b). While RyR2 clusters have been studied extensively in VMs, the distinct morphology of atrial RyR2 clusters and their organization in AT junctions warrants further investigation. Moreover, ultrastructural and close-to-native data about individual RyR2 clusters can inform direct structure-function analysis of individual clusters, for instance, how 3D spatial cluster arrangements control local Ca^2+^ release events as shown by mathematical modeling (Walker et al., 2014). Of note, a recent quantitative mathematical model of highly as well as minimally phosphorylated RyR2 clusters has reproduced AT-associated Ca^2+^ mega-sparks similar to biological Ca^2+^ signals observed in isolated AMs, and in line with the super-hub Ca^2+^ signaling concept (Brandenburg et al., 2016b). Future studies might employ the recently established STED Ca^2+^ imaging (Neef et al., 2018) to gain further experimental insight into the nanoscale Ca^2+^ signals at super-hub junctions at ATs.

In summary, live cell imaging by STED nanoscopy combined with customized membrane-preserving workflows and cholesterol-based staining has enabled direct visualization of AT structures throughout AMs. Furthermore, AM-specific TAT network structures were confirmed in atrial tissue sections of the rodent, porcine and human heart. Interestingly, based on collagenase perfusion, we consistently identified cell-wide TAT membrane networks in isolated AMs from mouse, rat, and rabbit hearts. Moreover, direct visualization of individual TAT components by STED nanoscopy showed abundant cholesterol nanodomains in AT membranes as well as local AT fragmentations in the absence of heparin pre-treatment. While AT membrane structures are abundant in AMs, they contain large LCC clusters and interact with extensive junctional RyR2 clusters. The nature of this complex super-hub protein-membrane assembly may support essential functions, in particular maintain and adapt atrial excitation-contraction coupling. Importantly, our finding of extensive junctional highphos RyR2 clusters will be instrumental to elucidate the differential nanodomain physiology and pharmacology of associated G protein-coupled receptors and ion channels associated with ATs. Notably, in samples from patients with chronic atrial fibrillation both LCC loss-of-function and RyR2 gain-of-function are now commonly observed (Vest et al., 2005; Schotten et al., 2011). In particular, RyR2 channel dysfunction was originally identified in the atria of elderly patients with chronic atrial fibrillation (Vest et al., 2005) and more recently after short-term tachypacing in rabbit atria (Greiser et al., 2014). Within this context, the causal relation between AT structures and highly phosphorylated junctional RyR2 clusters significantly strengthens recent rationales to identify early atria-specific disease mechanisms that may underlie atrial cardiomyopathies (Goette et al., 2017), and, ultimately, facilitate the development of new therapeutic rationales.

## Materials and Methods

### Mouse, Rat, and Rabbit Samples

Unless indicated otherwise, experiments were based on isolated AMs or atrial tissue from the hearts of different species: (a) 12–16 weeks old female mice in the C57BL/6N background; (b) 16–20 weeks old female Wistar rats; (c) female New Zealand White rabbits 17 weeks old with a body weight of 3.0 to 3.5 kg; and (d) 3-month-old healthy German landrace pigs of either gender with body weights of 40–45 kg. Mice and rats were euthanized by cervical dislocation under 1.5–2% isoflurane anesthesia; rabbits by pentobarbital (400 mg/kg). The generation of *Ryr2*^S2808A/S2808A^ knockin mice was described previously (Lehnart et al., 2005); for genotyping we used the following primer pairs: F 5′-ATCCCGAGGTAATCAGGTTTCT-3′, R 5′-AGTTGGGTTCAAAGTTCTAGGC-3′; and PCR product digestion by *Fsp I* restriction enzyme (New England Biolabs). This study was carried out according to guidelines for the care and use of laboratory animals following directive 2010/63/EU of the European Parliament and the Council of the European Union, Strasbourg, France, and in keeping with NIH guidelines. After mouse, rat, or rabbit euthanasia, the heart was rapidly extracted for atrial myocyte isolation (see below). All animal procedures followed institutional rules (Tötungsanzeige) as reviewed by the IACUC and the Veterinarian State Authority (LAVES, Oldenburg, Germany).

### Porcine Atrial Tissue Samples

Left atrial tissue was obtained from 3-month-old healthy German landrace pigs of either gender (*n* = 3, body weight 40–45 kg). Anesthesia was performed using azaperone, midazolam and propofol and isoflurane as reported (Schmidt et al., 2014). Pigs were sacrificed with intracardiac injection of KCl 7.45% and hearts were removed quickly. This study was performed in accordance with the Guide for the Care and Use of Laboratory Animals as adopted and promulgated by the United States National Institutes of Health (NIH publication No. 86-23, revised 1985), and the current version of the German Law on the Protection of Animals was followed (approval number G296/14).

### Human Atrial Samples

Human atrial tissue samples were obtained from the right atrial appendage of patients in sinus rhythm and a negative history for atrial fibrillation, undergoing open heart surgery for bypass grafting or valve replacement, respectively. Please see **Supplementary Table S1** for detailed clinical patient information. All patients gave written informed consent. The protocol to use human atrial samples was reviewed and approved by the ethics committee of the University Medical Center Göttingen (No. 14/9/11).

### Chol-PEG-KK114 Synthesis

The fluorescent Cholesterol analog Chol-PEG-KK114 was prepared by coupling the fluorescent dye KK114 (Kolmakov et al., 2010) to a Cholesterol-Polyethylene Glycol (Chol-PEG) derivative as follows. 4 mg (4 mM) of the dye-NHS ester were dissolved in dry *N*,*N*-dimethylformamide (DMF, 0.1 mL). A solution of 16 mg (6 μM) of Chol-PEG-NH2 (PG2-AMCS, NANOCS Inc., United States) in dry DMF (0.2 mL) and 5 μL (36 μM) trimethylamine were added and the reaction mixture was stirred for 40 min at room temperature. Then the reaction mixture was warmed up to 36°C and stirred for an additional 15 min. The reaction was controlled by thin-layer chromatography (TLC). After the volatile components were removed *in vacuo*, the product was isolated by flash column chromatography on SiO_2_ using Biotage Isolera flash purification system (Biotage SNAP Ultra 10 g cartridge; isocratic elution with 25% methanol and 5% water in chloroform). This yielded 5 mg (22%) of blue solid product. Analytical TLC was performed on ready-to-use silica gel 60 (F_254_) aluminum plates (Merck). Liquid chromatography (HPLC) was performed using a Knauer Smartline liquid chromatography system consisting of: two pumps (1000), a UV-detector 2500, a column thermostat 4000, a mixing chamber and injection valve with a 20 μL loop for the analytical column Eurospher 100 C18 (10 μm, 150 mm × 4 mm), and a 6-port-3-channel switching valve. A: acetonitrile + 0.1% v/v TFA, solvent B: H_2_O + 0.1% v/v TFA; temperature 25°C. HPLC: *t*_R_ = 9.2 min (A/B: 30/70 – 100/0 in 15 min, 1.2 ml/min, 254 nm). Mass spectra were recorded on a MICROTOF spectrometer (Bruker) equipped with an ESI ion source (Apollo) and a direct injector with the LC autosampler Agilent RR 1200. ESI-MS, negative mode: *m/z* (rel. int., %) = 1742.5 (40) ±*n* × 22 [*M*–3H+Na]^2-^, where *n* = 0–8 is the number of repeating ethylene oxide units OCH_2_CH_2_. The polydispersity of the copolymer reflects that of the PEG used in the synthesis.

### Atrial Myocyte Isolation From Mouse, Rat, and Rabbit Hearts

The AM isolation protocol used a modified Langendorff setup and cardiac perfusion starting with a nominally Ca^2+^ free buffer (in mM: NaCl 120.4, KCl 14.7, KH_2_PO_4_ 0.6, Na_2_HPO_4_ 0.6, MgSO_4_ 1.2, HEPES 10, NaHCO_3_ 4.6, taurine 30, 2,3-butanedione-monoxime 10, glucose 5.5, pH 7.4 with NaOH) followed by collagenase type II containing buffer for digestion of the extracellular matrix as previously described (Wagner et al., 2014; Brandenburg et al., 2016b). For mouse, rat, or rabbit hearts, 21 G, 17 G, or 14 G cannulas were used for aortic cannulation and perfusion at 4, 8, or 16 ml/min, respectively.

### Live Cell STED Nanoscopy

For superresolution imaging of TAT structures, isolated AMs were plated on laminin-coated coverslips and incubated for 10 min in perfusion buffer containing 5 μM Chol-PEG-KK114 for bulk membrane labeling. In addition, AMs were incubated for 15 min with 250 nM Chol-PEG-KK114 to visualize individual cholesterol-rich domains inside TAT structures. Following incubation, isolated AMs were washed three times in perfusion buffer and imaged at room temperature (20°C). A Ca^2+^ free perfusion buffer and 2,3-butanedione-monoxime were used to inhibit AM contractions. For imaging we used a Leica TCS SP8 STED system with a HC PL APO C2S 100×/1.40 oil objective. Microscope parameters were optimized for KK114: pixel size 16.23 nm × 16.23 nm, pixel dwell time of 400 ns, scanning speed 600 Hz, 32× line averaging, excitation using a white light laser at 635 nm, a STED depletion laser at 775 nm, and fluorescence detection at 650–700 nm. The STED laser power was adjusted to maximize resolution. In parallel, confocal imaging was applied for cell-wide TAT analysis at a pixel size of 114 nm × 114 nm with 16× line averaging. Raw images were processed in ImageJ/Fiji^1^. ROIs from 30 mouse AMs used for TAT network analysis in **Figure 3** are available for download through the online link: https://hdl.handle.net/21.11101/0000-0007-C9D2-9; doi: 10.5281/zenodo.1311573. Furthermore, images in **Figures 1B**, **4A,B** were deconvolved with Huygens Professional 17.10 software for visualization (Scientific Volume Imaging, Netherlands^2^). The fluorescence intensity plot profile of the cholesterol-rich nanodomain in **Figure 1B** was fitted by a 2D Gaussian function using OriginPro 8.5G followed by FWHM computation.

### TAT Network Analysis

2D skeletons were extracted from confocal live cell images of TAT structures stained with Chol-PEG-KK114 using ImageJ/Fiji as previously described (Brandenburg et al., 2016b). Cells were aligned and ROIs were selected excluding the surface membrane and nuclei. After background subtraction and smoothing, ROIs were binarized using a predefined threshold and consecutively skeletonized. Network length and the number of network junctions were calculated in ImageJ/Fiji using the plugin Analyze Skeleton (2D/3D). TAT network component orientations were analyzed via the plugin Directionality.

### Tubule Diameter Measurements

Chol-PEG-KK114 stained STED images of ATs and TTs were aligned. ROIs of 50 pixels × 30 pixels (pixel size 16.23 nm × 16.23 nm) were manually selected and used for fluorescence intensity plot profiling. AT and TT signal profiles were fitted by a 2-peak Gaussian function using OriginPro 8.5G to calculate FWHM as a measure of tubule width.

### Histology of Atrial Tissue

For immunofluorescence labeling, atrial tissues from rat, rabbit, pig, and human were fixed in 4% PFA over night, embebbed in paraffin, cut into 4 μm thick histological sections, deparaffinized, rehydrated, and the antigen unmasked with 10 mM sodium-citrate buffer prior to WGA or antibody incubation.

### Immunofluorescence STED Nanoscopy

Isolated cardiomyocytes were plated on laminin-coated coverslips and fixed in 4% PFA for 5 min followed by three PBS washing steps. Next, isolated myocytes or unmasked histological tissue sections were incubated in blocking/permeabilization buffer over night (10% bovine calf serum, 0.2% Triton in PBS). Primary antibodies were diluted in blocking buffer and incubated over night at 4°C as follows: RyR2 1:500 (HPA020028, Sigma-Aldrich); RyR2 1:500 (MA3-916, Thermo Fisher Scientific); RyR2-pS2808 1:250 (A010-30, Badrilla Ltd.); Caveolin3 1:500 (610421, BD Biosciences); Ca_v_1.2 1:250 (ACC-003, Alomone labs); Ca_v_1.3 1:100 (ACC-005, Alomone labs). For histological sections, the Caveolin3 antibody was used at a dilution of 1:250. Subsequently, samples were washed three times in blocking buffer and incubated with secondary antibodies diluted 1:1000 for cells or 1:300 for tissue sections for 2 h at room temperature: anti-rabbit (STAR 635P, Abberior) and anti-mouse (STAR 580, Abberior). Unconjugated WGA (VEC-L-1020-10, Biozol) was labeled by NHS ester (STAR 635P, Abberior; 07679, Sigma-Aldrich) and incubated at a concentration of ≈10 μM with samples in blocking buffer for 2 h in combination with the secondary anti-mouse antibody (STAR 580, Abberior). After washing, samples were embedded in mounting medium (ProLong Gold antifade reagent, Thermo Fisher Scientific). A Leica TCS SP8 STED microscope was used for superresolution imaging using the parameters stated above. STAR 635P and STAR 580 fluorophore emission was detected between 650–700 nm and 600–630 nm, respectively. Raw images were processed in ImageJ/Fiji.

### Immunohistochemistry of Inner Hair Cells

Apical cochlear turns were fixed in methanol for 20 min at −20°C immediately after dissection. The tissue was then washed three times for 10 min in PBS and incubated for 1 h in goat serum dilution buffer (GSDB) (16% normal goat serum, 450 mM NaCl, 0.3 Triton X-100, 20 mM phosphate buffer, pH 7.4) in a wet chamber at room temperature. Primary antibodies (mouse anti-Sap7f407 to bassoon, 1:600, Abcam ab82958, and rabbit anti-Ca_v_1.3, 1:75, Alomone Labs ACC-005) were diluted in GSDB and applied overnight at 4°C in a wet chamber. After washing three times for 10 min (wash buffer: 450 mM NaCl, 20 mM phosphate buffer, and 0.3% Triton X-100), the tissue was incubated with secondary antibodies [STAR 580-tagged goat-anti-rabbit or goat-anti-mouse (1:200, Abberior 2-0002-005-1 or 2-0012-005-8)], and STAR 635P-tagged goat-anti-mouse or goat-anti-rabbit (1:200, Abberior 2-0002-007-5 or 2-0012-007-2) in GSDB in a wet light-protected chamber for 1 h at room temperature. The preparations were then washed three times for 10 min in wash buffer and one time for 10 min in 5 mM phosphate buffer, placed onto glass microscope slides with a drop of fluorescence mounting medium (Mowiol), and covered with thin glass coverslips. Images were acquired on an Abberior Instruments Expert Line 775 nm 2-color STED microscope, with excitation lasers at 561 and 633 nm and a STED laser at 775 nm, 1.2 W, using a 1.4 NA 100× oil immersion objective, with pixel sizes of 20 nm × 20 nm.

### Random Access Multi-Photon Microscopy (RAMP)

Isolated cardiomyocytes were loaded in Tyrode’s solution with 10 μM of blebbistatin and 4 μM of cytochalasin D both dissolved in DMSO. 2 μg/mL of di-4-AN(F)EPPTEA (Yan et al., 2012) dissolved in ethanol were added for 15 min, and then cells were resuspended in fresh Tyrode’s buffer containing blebbistatin and cytochalasin D. Loaded AM preparations were used for RAMP experiments within 30 min. The staining and imaging sessions were performed at room temperature. The RAMP imaging system has been described previously in detail (Sacconi et al., 2012; Crocini et al., 2014, 2016). Briefly, a 1064 nm fiber laser provides the excitation light. The scanning head is provided through two orthogonally oriented acousto-optic deflectors (AODs) and the excitation light is focused onto the specimen by the objective lens (60 × 1.4 NA). The two-photon fluorescence signal is collected forward and backward by an oil immersion condenser (1.4 NA) and the objective, respectively. Fluorescence signals are detected by two photomultiplier tubes (H7422, Hamamatsu) using an emission filter of 655 ± 20 nm. Measurements are performed during steady-state stimulation (0.34 Hz). Cells are field-stimulated using two parallel platinum wires (250 μm in diameter) placed at a distance of 6.3 mm. Square pulses of 10–20 V and duration of 3 ms were used to reach action potential threshold. In a typical measurement, we sampled 5–6 different sarcolemmal sites through 10 subsequent trials. The length of the scanned lines ranges from 2 to 10 μm with an integration time per membrane pass of ∼100–200 μs, leading to a temporal resolution of 0.5–1.5 ms.

### Combined Imaging of TAT Membranes and Intracellular Ca^2+^

Transverse line scan imaging of AMs plated on laminin-coated coverslips was performed with a Zeiss LSM 880 confocal microscope and a Plan-Apochromat 63×/1.40 oil objective. The membrane dye Chol-PEG-KK114 (10 min incubation of the cells at 1 μM) and the Ca^2+^ indicator Fluo-4 AM (30 min incubation of the cells at 10 μM; Thermo Fisher Scientific) signals were recorded as previously described (Brandenburg et al., 2016b) at room temperature in Tyrode’s solution (in mM: NaCl 140, KCl 5.4, MgCl_2_ 1.2, HEPES 10, Na_2_HPO_4_ 0.33, CaCl_2_ 1, glucose 10; pH 7,4 with NaOH). Chol-PEG-KK114 was excited at 633 nm and detected between 650 and 700 nm. AMs were field-stimulated at 0.5 Hz using 3 ms steps to 23 V. Transverse lines of 20 μm (100 pixels) were scanned at 650 Hz. ImageJ/Fiji was used for image processing.

### Combined Patch-Clamp and Intracellular [Ca^2+^] Measurements

Human AMs were isolated from right atrial tissue samples using protocols established previously (Voigt et al., 2013, 2014). Please see **Supplementary Table S1** for clinical patient information. Membrane currents were measured at 37°C in whole-cell ruptured-patch configuration using voltage-clamp with simultaneous intracellular [Ca^2+^] measurements. pClamp-Software (V10.7, Molecular Devices, Sunnyvale, CA, United States) was used for data acquisition and analysis. Intracellular [Ca^2+^] was quantified using 10 μM of the Fluo-3-acetoxymethyl ester (Fluo-3 AM; Invitrogen, Carlsbad, CA, United States) with 10 min loading and 30 min de-esterification (Grynkiewicz et al., 1985). In addition, Fluo-3 was included into the electrode solution containing (in mM): EGTA 0.02, Fluo-3 0.1 (Invitrogen), GTP-Tris 0.1, HEPES 10, K-aspartate 92, KCl 48, Mg-ATP 1, Na_2_-ATP 4; pH7.2. Borosilicate glass microelectrodes had tip resistances of 2–4 MΩ when filled with the pipette solution. Seal-resistances were 4–8 GΩ. For voltage-clamp experiments, the series resistance and cell capacitance were compensated. AMs were superfused at 37°C with a bath solution containing (in mM): CaCl_2_ 1, glucose 10, HEPES 10, KCl 4, MgCl_2_ 1, NaCl 140, probenecid 2, pH7.4. K^+^ currents were blocked by 4-aminopyridine (5 mM) and BaCl_2_ (0.1 mM) in the bath solution. L-type Ca^2+^-current (*I*_Ca,L_) and corresponding [Ca^2+^]_i_ transients were recorded simultaneously, using a holding potential of −80 mV and ramp pulse depolarization to −40 mV for 500 ms in mouse AMs or 100 ms in human AMs to inactivate the fast Na^+^-current followed by a 100 ms test pulse to +10 mV at 0.5 Hz. Membrane current amplitude and integrated membrane current were normalized to cell capacitance as a measure of the cell surface area for atrial myocytes from different species. The sarcoplasmic reticulum (SR) Ca^2+^ content was determined by the application of 10 mM caffeine at a holding potential of −80 mV, with the resulting NCX current integrated and normalized to capacitance measurements from cells. Corresponding diastolic calcium removal mechanisms, i.e., SERCA and non-SERCA flux components (NCX, PMCA, and mitochondria) were mathematically interpolated from caffeine induced [Ca^2+^]_i_ transients and systolic [Ca^2+^]_i_ transients as described.

### Protein Analysis

For protein analysis, mouse and rat samples were selected sex-mixed. Atrial tissue was directly frozen in liquid nitrogen and stored at −80°C. Then, atrial tissues were homogenized in ice-cold homogenization buffer [HEPES 10 mM, sucrose 300 mM, NaCl 150 mM, EGTA 1 mM, CaCl_2_ 2 mM, Triton X-100 0.5% (v/v), protease and phosphatase inhibitor mix (Roche), pH 7.4] using a Miccra D-1 homogenizer. The homogenized tissues were solubilized for 30 min at 4°C by rotation and subsequently centrifuged at 8000 × *g* for 10 min at 4°C to obtain the post-nuclear fraction. Protein concentrations were determined with the Pierce BCA protein Assay Kit (Thermo Fisher Scientific). For Western blots 20 μg of protein per lane were resolved by SDS-PAGE using 4–20% Tris-HCl protein gels (3450033, Bio-Rad). Proteins were transferred onto PVDF membranes (0.45 μm, Immobilon-FL, Merck Millipore) using the Bio-Rad criterion blotter (plate electrodes). Membranes were blocked for 1 h in 5% (w/v) non-fat milk in Tris-buffered saline with 0.05% (v/v) Tween 20, and incubated with the primary antibodies overnight at 4°C as follows: RyR2 1:2500 (HPA020028, Sigma-Aldrich); NCX 1:1000 (11-13, Swant); SERCA2 1:2000 (A010-20, Badrilla); Na,K-ATPase α1 subunit 1:500 (sc-21712, Santa-Cruz); GAPDH 1:160,000 (5G4 Mab 6C5, HyTest); PLN 1:2500 (ab2865, Abcam); PLM 1:1000 (13721-1-AP, Proteintech). After washing, blots were incubated with fluorescence-labeled anti-rabbit or anti-mouse secondary antibodies at a dilution of 1:10,000 for a minimum period of 1 h at room temperature (LI-COR, P/N 926-32212, P/N 926-68072, P/N 926-32213, P/N 926-68073). Membranes were developed with the Odyssey CLx imaging system (LI-COR). Band densitometry analysis was performed using Image Studio Lite Version 5.2 and normalized to GAPDH.

### Statistics

Statistical analyses were performed with Microsoft Excel 2010, GraphPad Prism 7.03, and SigmaPlot 12.3. All data are normally distributed and presented as the mean ± the standard error of the mean (SEM). *P*-values < 0.05 were accepted to indicate statistical differences.

## Data Availability

ROIs from 30 mouse AMs analyzed for TAT network analysis in **Figure 3** are available for download via online link: https://hdl.handle.net/21.11101/0000-0007-C9D2-9; doi: 10.5281/zenodo.1311573. Raw data supporting the conclusions of this manuscript will be made available by the authors, without undue reservation, to any qualified researcher.

## Author Contributions

SB and SEL were responsible for the central hypothesis, design and implementation of the study, collection and analysis of experimental data, and for preparing the manuscript. CS, DK-D, FF, FP, FW, IK, JN, JP, LS, MS, NV, SB, SEL, SS, and TK performed experiments, analyzed data, and contributed to the manuscript. IK, NV, and SS provided expertise about human heart samples and data analysis, and contributed to the manuscript. GM contributed expertise for customized synthesis and quality control of fluorescent lipid markers and contributed to the manuscript. TM provided critical comments, data, and analysis for the manuscript.

## Conflict of Interest Statement

The authors declare that the research was conducted in the absence of any commercial or financial relationships that could be construed as a potential conflict of interest.

## Footnotes

1 http://imagej.nih.gov/ij/

2 http://svi.nl
