# Supplementary material for: Axial Tubule Junctions Activate Atrial Ca2+ Release Across Species
Source: Front Physiol. 2018 Oct 8;9:1227. doi: 10.3389/fphys.2018.01227 (PMC6187065; doi:10.3389/fphys.2018.01227)
Supplement: Supplementary file 1 [file Image_1.pdf]

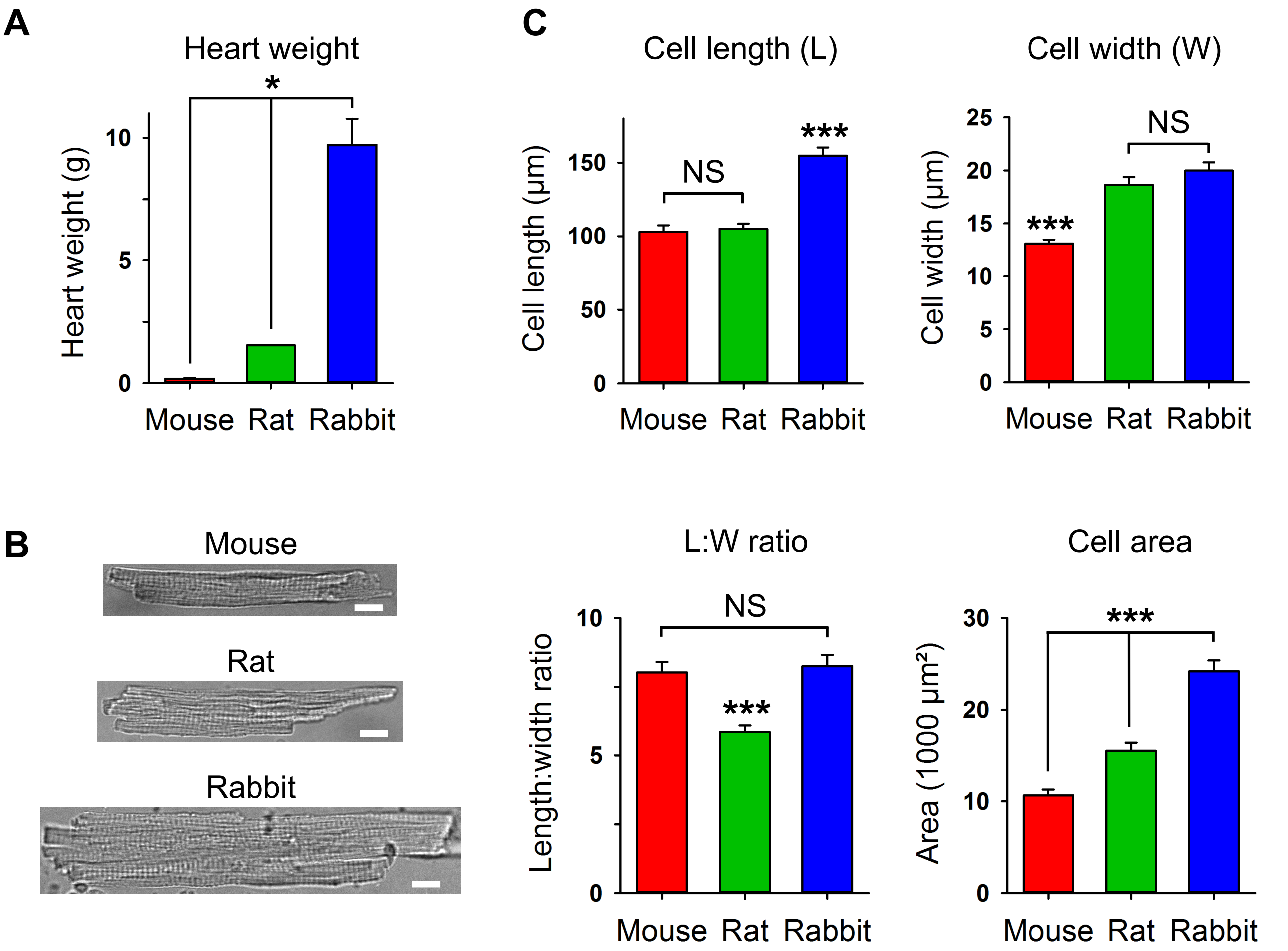

Supplementary Figure 1. Atrial cell and heart dimensions in adult mouse, rat and rabbits. (**A**) Bar graph comparing adult heart weights for mouse, rat, and rabbit. n = 3 hearts for each species. (**B**) Representative bright field images of isolated AMs from mouse, rat and rabbit hearts. Note the intact surface boundaries and intracellular sarcomere striations. Scale bars 10 µm. (**C**) Length, width, length/width ratio and calculated area of live atrial myocytes. Mouse n = 28; rat n = 35; and rabbit n = 47 atrial myocytes from 3 hearts each. For analysis, cell surface boundaries were selected manually from the ROIs of each cell's bright field image using ImageJ/Fiji. * $P < 0.05$, *** $P < 0.001$, by Student's t-test.
