# Supplementary material for: Axial Tubule Junctions Activate Atrial Ca2+ Release Across Species
Source: Front Physiol. 2018 Oct 8;9:1227. doi: 10.3389/fphys.2018.01227 (PMC6187065; doi:10.3389/fphys.2018.01227)
Supplement: Supplementary file 2 [file Image_2.pdf]

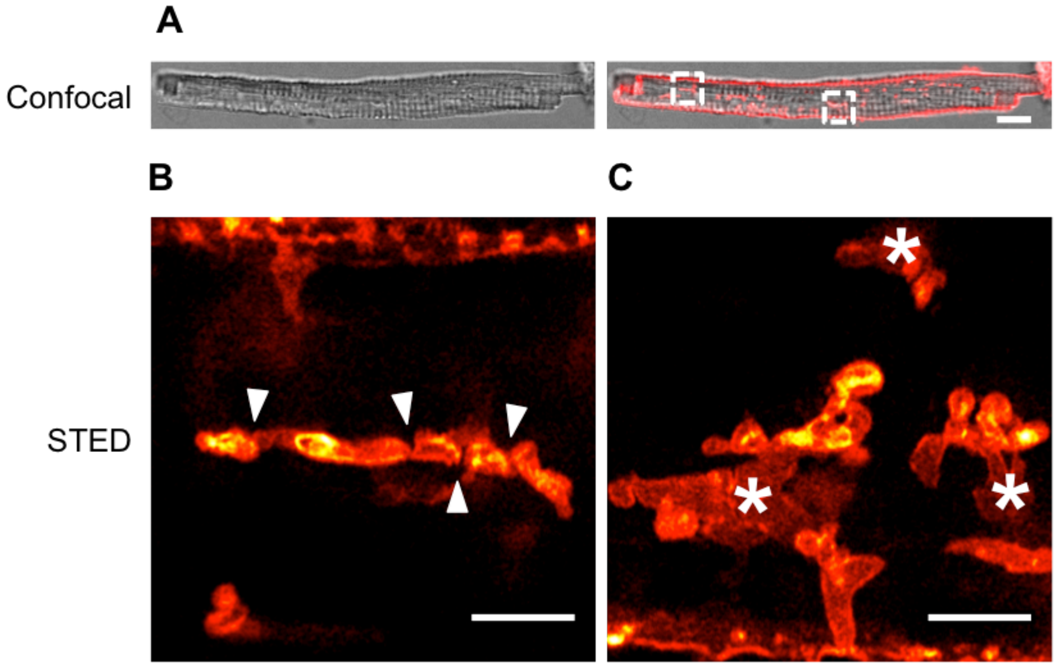

Supplementary Figure 2. Axial tubule fragmentation and membranous aggregation in living rabbit atrial myocytes after isolation without heparin. Bright field view and confocal image superposition showing the TAT network in a living rabbit atrial myocyte using the membrane dye Chol-PEG-KK114. Despite confocal resolution, membrane injuries are not apparent (**A**). In contrast, in the same rabbit atrial myocyte STED nanoscopy resolves numerous axial tubule fragmentation events (white arrowheads) (**B**) and large membranous aggregates containing tubule components (asterisks) (**C**). Dashed boxes indicate magnified regions shown in (**B**) and (**C**). N, nucleus. Scale bars 10 µm (**A**); 2 µm in (**B**) and (**C**).
