# Supplementary material for: Axial Tubule Junctions Activate Atrial Ca2+ Release Across Species
Source: Front Physiol. 2018 Oct 8;9:1227. doi: 10.3389/fphys.2018.01227 (PMC6187065; doi:10.3389/fphys.2018.01227)
Supplement: Supplementary file 3 [file Image_3.pdf]

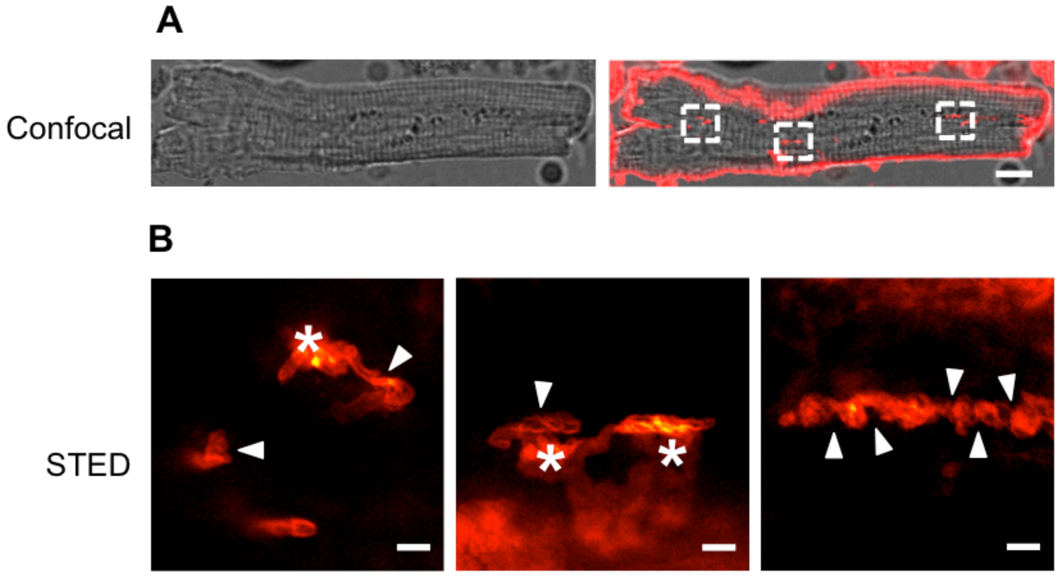

Supplementary Figure 3. Axial tubule fragmentation and membranous aggregation in isolated human atrial myocytes. (A) Bright field view and superimposed confocal image based on bulk membrane labeling with Chol-PEG-KK114 in isolated human atrial myocytes. **(B)** The mechanical dissociation and isolation of human atrial myocytes leads to TAT network fragmentation. Residual axial tubule components show fragmentation events (arrowheads) and membranous aggregates (asterisks) and disruption of the TAT network as resolved by STED nanoscopy. Dashed boxes in (**A**) indicate regions magnified in (**B**). Scale bars 10 µm in (**A**); 1 µm in (**B**).
