# Supplementary material for: Axial Tubule Junctions Activate Atrial Ca2+ Release Across Species
Source: Front Physiol. 2018 Oct 8;9:1227. doi: 10.3389/fphys.2018.01227 (PMC6187065; doi:10.3389/fphys.2018.01227)
Supplement: Supplementary file 4 [file Image_4.pdf]

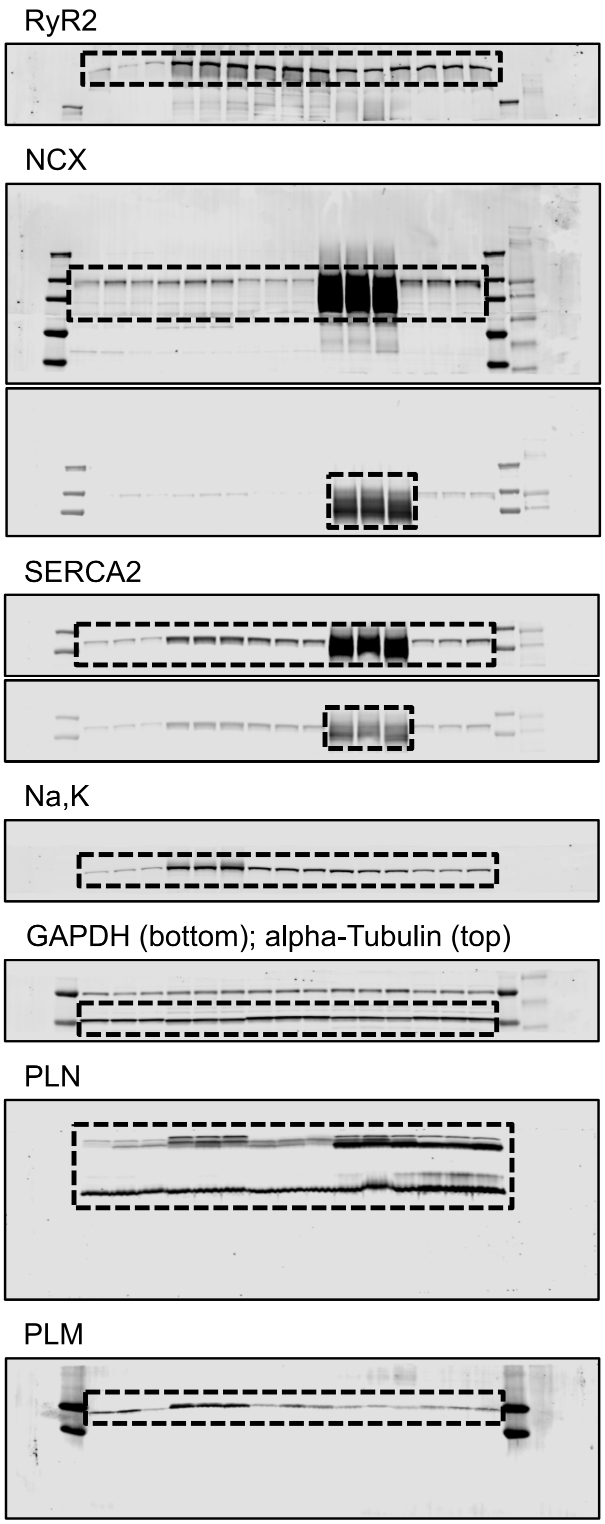

Supplementary Figure 4. Full scans of the Western blots reported. Dashed boxes indicate the data presented in **Figure 7**.
