# Supplementary material for: Axial Tubule Junctions Activate Atrial Ca2+ Release Across Species
Source: Front Physiol. 2018 Oct 8;9:1227. doi: 10.3389/fphys.2018.01227 (PMC6187065; doi:10.3389/fphys.2018.01227)
Supplement: Supplementary file 5 [file Image_5.pdf]

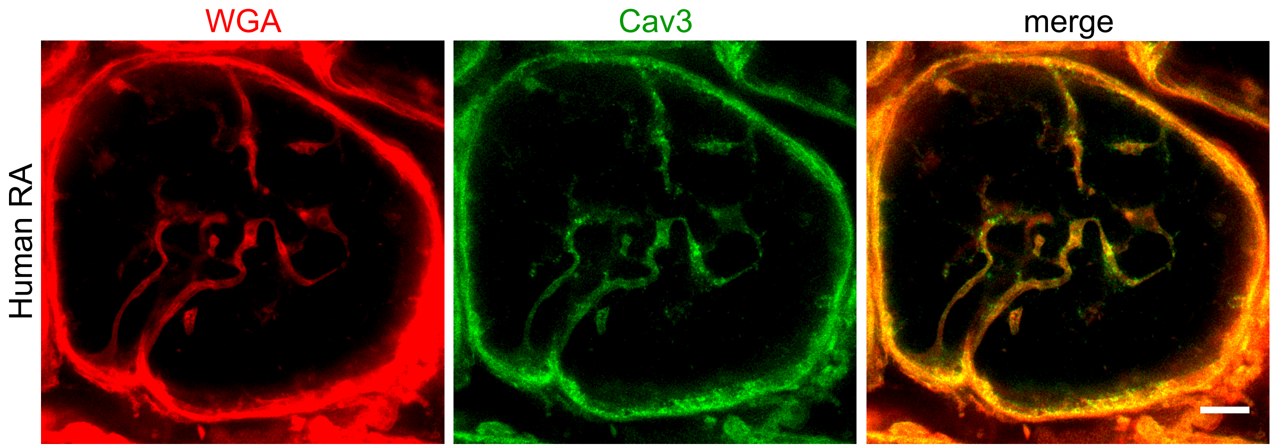

Supplementary Figure 5. Transverse tubule invaginations penetrate from the surface sarcolemma deep into the cell center of human atrial myocytes. STED imaging based on WGA and anti-Cav3 co-staining of 4 µm thick histological sections of human right atrial tissue. The STED images show an example of a transverse section through a human atrial myocyte. Scale bar 2 µm.
