# Supplementary material for: Axial Tubule Junctions Activate Atrial Ca2+ Release Across Species
Source: Front Physiol. 2018 Oct 8;9:1227. doi: 10.3389/fphys.2018.01227 (PMC6187065; doi:10.3389/fphys.2018.01227)
Supplement: Supplementary file 6 [file Table_1.pdf]

	Sinus rhythm
Patients, n	13
Gender, m/f	11/2
Age, y	61.9±3.6
Body mass index, kg/m^2	27.0±1.0
CAD, n	5
MVD/AVD, n	6
CAD+MVD/AVD, n	2
Hypertension, n	8
Diabetes, n	2
Hyperlipidemia, n	5
LVEF, %	54.0±1.7
Digitalis, n	0
ACE inhibitors, n	6
AT1 blockers, n	2
β-Blockers, n	8
Dihydropyridines, n	3
Diuretics, n	4
Nitrates, n	0
Lipid-lowering drugs, n	8

Supplementary Table 1. Clinical patient information. Human atrial tissue samples were obtained from the right atrial appendage of patients in sinus rhythm (negative history for atrial fibrillation), undergoing open heart surgery for bypass grafting or valve replacement, respectively. Data for age, body mass index and LVEF are presented as mean ± SEM. CAD, coronary artery disease; MVD/AVD, mitral/aortic valve disease; LVEF, left ventricular ejection fraction; ACE, angiotensin-converting enzyme; AT, angiotensin receptor.
